# Nanostructured Silicon Anodes for Lithium-Ion Batteries: Advances, Challenges, and Future Prospects

**DOI:** 10.3390/ma19020281

**Published:** 2026-01-09

**Authors:** Alexander A. Pavlovskii, Konstantin Pushnitsa, Alexandra Kosenko, Pavel Novikov, Anatoliy A. Popovich

**Affiliations:** Institute of Machinery, Materials and Transport, Peter the Great Saint Petersburg Polytechnic University (SPbPU), Politechnicheskaya ul. 29, 195251 Saint Petersburg, Russianovikov.p.a@gmail.com (P.N.); popovicha@mail.ru (A.A.P.)

**Keywords:** silicon anode, lithium-ion battery, silicon nanoparticles, porous silicon

## Abstract

Silicon is considered one of the most promising next-generation anode materials for lithium-ion batteries (LIBs) because of its very high theoretical specific capacity (≈3579 mAh·g^−1^). However, its practical application is limited by severe volume expansion (>300%), an unstable solid electrolyte interphase (SEI), and low electronic conductivity. Recent progress in nanostructuring has significantly improved the electrochemical performance and durability of silicon anodes. In particular, nanosilicon particles, porous structures, and Si–carbon composites enhance structural stability, cycling life, and coulombic efficiency. These improvements arise from better mechanical integrity and more stable electrode–electrolyte interfaces. This review summarizes recent advances in nanostructured silicon anodes, focusing on particle size control, pore design, composite architectures, and interfacial engineering. We discuss how these nanoscale strategies reduce mechanical degradation and improve lithiation kinetics while also addressing the remaining challenges. Finally, future research directions and industrial prospects for the practical use of nanostructured silicon anodes in next-generation LIBs are outlined.

## 1. Introduction

The global demand for high-energy, long-cycle-life lithium-ion batteries continues to increase rapidly due to the rapid expansion of electric vehicles, grid-scale renewable energy storage, and portable electronics [1,2,3]. While commercial graphite anodes provide stable cycling performance and low cost, their practical use is fundamentally restricted by a limited specific capacity of 372 mAh·g^−1^ [4]. Silicon, possessing an exceptionally high theoretical capacity of approximately 3579 mAh·g^−1^ [5], has long been regarded as one of the most promising next-generation anode materials.

However, silicon suffers from substantial volumetric expansion of up to 300% during lithiation, which results in severe mechanical stresses, particle fracture, loss of electrical contact, and repeated formation and breakdown of the SEI. These degradation processes collectively cause rapid capacity fading and poor cycling stability [6,7]. In addition, the spontaneous formation of a native oxide layer and the intrinsically sluggish kinetics of lithium–silicon alloying further hinder the reversibility of lithiation and contribute to significant irreversible capacity losses [8].

Nanostructuring strategies offer a transformative pathway to overcoming these intrinsic limitations. Reducing silicon to the nanoscale enables more effective strain accommodation, mitigates mechanical pulverization, preserves continuous electrical contact, and enhances Li-ion transport kinetics [9,10]. A wide range of nanoscale architectures has been developed, including hollow nanoparticles [10], yolk–shell designs [11], porous silicon frameworks (including biomass-derived Si [12,13]), graphene-confined silicon [14], and hybrid silicon–carbon architectures [15,16]. Collectively, these designs significantly improve structural resilience and electrochemical performance.

Silicon anodes engineered with low-dimensional architectures, such as zero-dimensional (0D) silicon nanoparticles (Si NPs), one-dimensional (1D) nanowires, nanofibers, and nanotubes, two-dimensional (2D) silicon thin films, as well as three-dimensional (3D) porous or nanoporous silicon frameworks, exhibit several key advantages over bulk silicon electrodes. These benefits arise from their enlarged surface area, accelerated electron transport, shortened lithium diffusion pathways, and improved structural tolerance to volume changes during lithiation and delithiation. Furthermore, the high density of grain boundaries present in nanoscale silicon provides additional diffusion channels and supplementary lithium-storage sites.

Such nanostructured silicon materials represent up-and-coming anode candidates for next-generation lithium-ion batteries because their engineered geometries can mitigate electrode pulverization, suppress particle fragmentation, and enhance the stability of the solid electrolyte interphase. The synthesis of these materials commonly relies on the principle of spatial or structural stabilization, wherein the creation of internal free volume, such as pores, voids, or hollow regions, enables the accommodation of the expanding Li_x_Si alloy formed during charging. This internal buffering significantly reduces the detrimental mechanical stresses associated with silicon’s significant volumetric changes.

Importantly, the concept of low-dimensional silicon was validated early on through thin-film silicon studies, in which excellent cycling stability was consistently demonstrated. Prior investigations revealed that electrodes with reduced silicon film thickness exhibit superior capacity retention, and that higher charging currents can further enhance the durability of such thin-film architectures [7,17].

Beginning in 1994, pioneering work by Wilson and Dahn at Simon Fraser University initiated the development of nanosilicon embedded within carbon matrices [18]. Although silicon constituted only a minor fraction of these composites, their studies convincingly demonstrated that nanoscale silicon could function as a viable room-temperature anode, ultimately providing the foundation for a new generation of nanostructured silicon designs. Shortly thereafter, rapid advancements followed. In 1998, Wang and co-workers at Zhejiang University produced silicon nanoparticles via high-energy ball milling without the use of a carbon matrix [19], and in 1999, Li and collaborators at the Chinese Academy of Sciences prepared silicon nanoparticles using a molecular precursor route [20]. Both groups reported reversible lithiation–delithiation behavior at ambient conditions, further validating the promise of nano-Si as an anode material.

Between 2000 and 2010, research on silicon anodes accelerated substantially. Numerous studies from leading academic groups revealed that strategies such as particle nanosizing, amorphization, and controlled restriction of volume expansion could dramatically improve electrode lifetime. Several breakthroughs, including silicon nanoparticles and silicon nanowires, eventually contributed to the formation of commercial ventures such as Amprius and Sila Nanotechnologies, which continue to pursue large-scale deployment of silicon-based anode technologies.

Despite these advancements, a major barrier in nanosilicon research remains the high cost associated with nanoparticle synthesis and post-processing. Commercial Si nanoparticles are priced disproportionately high, even though silicon is the second most abundant element in Earth’s crust. This discrepancy arises from the fact that naturally occurring silicon is found predominantly as silica or silicate minerals, making extraction of elemental silicon energy-intensive and expensive. Consequently, there is a strong need for scalable, economical synthesis approaches that can produce nanosilicon with controlled sizes, morphology, and properties suitable for next-generation lithium-ion battery anodes.

This review provides a comprehensive overview of advanced nanostructured silicon anodes, covering their degradation mechanisms, synthesis techniques, classification of nanoscale architectures, electrochemical performance, key challenges, and emerging strategies to facilitate their large-scale commercialization.

## 2. Classification of Silicon Nanostructures by Dimensionality (0D–3D)

Nanostructuring silicon has emerged as one of the most effective strategies for overcoming its intrinsic mechanical instability during lithiation and delithiation. Among the various design approaches, classifying silicon architectures by dimensionality, from zero-dimensional (0D) nanoparticles to three-dimensional (3D) porous frameworks, provides a systematic basis for understanding how morphology governs electrochemical behavior. Each dimensionality class offers distinct pathways for mitigating volumetric expansion, enhancing ion and electron transport, and stabilizing the SEI.

0D structures minimize diffusion lengths and allow highly uniform strain distribution; 1D architectures provide continuous electron-conducting channels; 2D thin films suppress fracture through constrained geometries; and 3D porous or hollow frameworks introduce internal free volume capable of buffering expansion. These engineered geometries also enable diverse synthetic routes, ranging from magnesiothermic reduction and templating methods to bottom-up chemical synthesis and biomass-derived processes.

In the following sections, silicon nanostructures are systematically categorized according to dimensionality, highlighting their fabrication strategies, electrochemical characteristics, and relevance to high-performance lithium-ion battery anodes.

### 2.1. Zero-Dimensional (0D) Silicon Structures

Here we highlight the specific role and importance of 0D structures, such as silicon nanoparticles and their carbon-modified derivatives. Owing to their extremely short lithium-ion diffusion pathways and the ability to distribute strain more uniformly, 0D Si architectures are especially effective in addressing two critical issues: severe volume changes during cycling and limited intrinsic conductivity. In this subsection, we present a detailed overview of recent advances in 0D silicon design, fabrication strategies, and composite engineering, demonstrating how these nanoscale configurations contribute to improved performance and long-term stability of silicon-based lithium-ion battery anodes.

Silicon nanoparticles, representing the class of zero-dimensional silicon materials, have emerged as highly attractive candidates for lithium-ion battery anodes for two key reasons. First, their nanoscale dimensions help mitigate mechanical stress and suppress structural degradation during lithiation and delithiation, thereby improving cycling stability [21,22]. Second, synthesis routes for Si nanoparticles are mature and scalable, contributing to their growing commercial availability [23].

Porous and hollow Si nanoparticles generally offer superior electrochemical performance due to several beneficial features:

1. Internal voids and porosity accommodate volume expansion, protecting the material from structural failure during repeated charge–discharge cycles and enabling long-term durability.
2. The shortened lithium-ion and electron diffusion paths reduce polarization and enhance rate capability [24], allowing rapid energy storage and delivery.
3. Reduced local current density in porous structures minimizes stress gradients at particle surfaces, improving the mechanical and electrochemical stability of the electrode.

#### 2.1.1. Classical Synthetic Routes and Representative Examples

**Figure 1:**
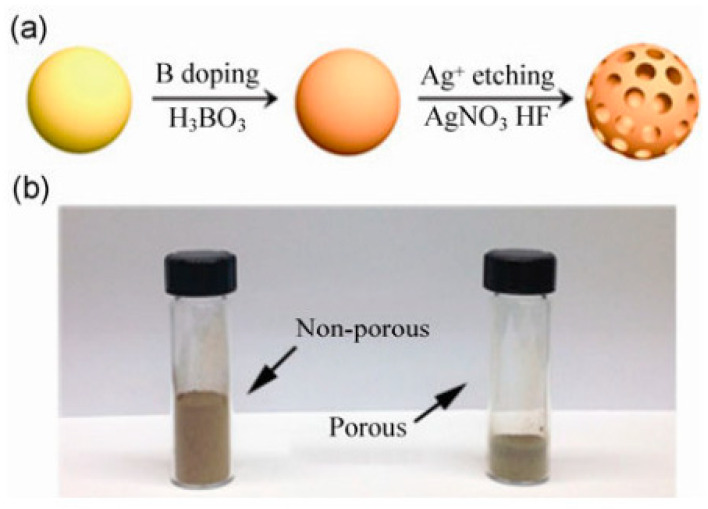
(**a**) Schematic representation of the synthesis procedure for porous silicon nanoparticles. (**b**) Visual comparison between pristine nonporous silicon nanoparticles (**left**) and the resulting porous silicon nanoparticles (**right**), highlighting the effectiveness and scalability of the fabrication method. Reprinted from Ref. [25], Copyright 2013, with permission from Springer Nature.

Chemical etching combined with self-assembly has been widely employed to produce porous silicon nanoparticles. Ge and co-workers [25] prepared porous Si nanoparticles through electroless etching followed by boron doping. When integrated with graphene, the resulting composite delivered a stable capacity of 1000 mAh·g^−1^ after 200 cycles. The synthesis strategy and the structural characteristics of the obtained porous silicon are schematically illustrated in Figure 1. As shown in Figure 1a, the electroless etching route enables controlled pore formation through a simple and scalable process. The comparison in Figure 1b between commercial nonporous silicon nanoparticles and their porous counterparts directly demonstrates the generation of internal void space, which is critical for buffering silicon volume expansion and preserving structural integrity during repeated lithiation–delithiation cycles.

Similarly, Wang and co-workers [26] developed a mesoporous Si/C composite obtained via an evaporation-induced self-assembly process using a triblock copolymer in a resorcinol–formaldehyde resin. This method produced a uniform dispersion of ~100 nm Si nanoparticles within a mesoporous carbon matrix, delivering a capacity of 1018 mAh·g^−1^ over 100 cycles.

Carbon nanotube–based architectures have also shown promise for mitigating silicon’s volume expansion. Epur and collaborators [27] designed a binder-free Si/MWCNT anode architecture that reached an impressive initial discharge capacity of 3112 mAh·g^−1^ at 300 mA·g^−1^. Notably, the electrode retained 76% of this capacity after 50 cycles, a performance attributed to the strong CNT–Si interfacial connections that minimized volume fluctuations and ensured efficient electronic conduction.

In a similar approach, Liu and colleagues [28] proposed a highly effective strategy for synthesizing Cu_2_MoS_4_/SiNS composites through the self-assembly of silicon nanospheres with two-dimensional Cu_2_MoS_4_ sheets. The composite exhibited a well-defined attachment of Si nanospheres onto the sheet-like Cu_2_MoS_4_ structure. TEM imaging confirmed the presence of porous silicon uniformly integrated within the layered matrix, a configuration that helps accommodate volume expansion, alleviate mechanical stress, and provide additional active sites and fast Li^+^ transport pathways. When evaluated as an anode material, the Cu_2_MoS_4_/SiNS composite demonstrated significantly enhanced electrochemical behavior. Under fast-charging conditions at 2.0 A·g^−1^, it delivered a specific capacity of 1180 mAh·g^−1^ with 69.2% capacity retention after 400 cycles. The outstanding stability and high capacity of this composite highlight its potential for next-generation high-performance lithium-ion batteries.

**Figure 2:**
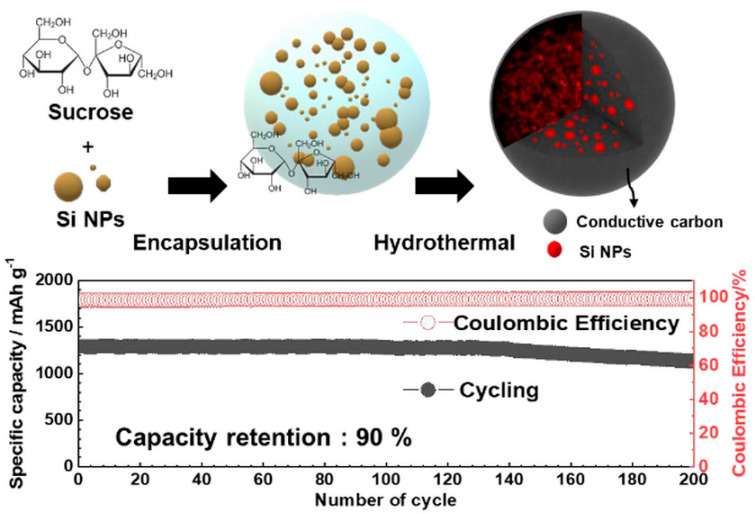
Schematic illustration of the one-pot hydrothermal synthesis of the meso–macroporous Si–C composite, showing 40 wt.% Si nanoparticles embedded within carbon spheres (~3 μm) with hierarchical porosity (**top**), and electrochemical performance of the composite anode in lithium-ion cells, demonstrating an initial capacity of 1300 mAh·g^−1^, 90% capacity retention after 200 cycles, and corresponding Coulombic efficiency (**bottom**). Reprinted with permission from Ref. [29], Copyright 2020, American Chemical Society.

Hydrothermal strategies have been applied to prepare Si–carbon composites with hierarchical porosity. Jung’s research team [29] synthesized a Si–carbon composite via a one-pot hydrothermal process, in which surface-etched Si nanoparticles were combined with sucrose as the carbon source. The resulting meso–macroporous Si–C material incorporated a substantial Si nanoparticle content (40 wt.%) confined within carbon spheres approximately 3 μm in diameter, forming a hierarchical porous architecture (Figure 2, top). As illustrated in Figure 2 (top), the confinement of Si nanoparticles within meso–macroporous carbon spheres creates internal void space and continuous conductive pathways, which effectively accommodate silicon volume changes while preserving electrical connectivity. Correspondingly, the electrochemical data shown in Figure 2 (bottom) demonstrate an initial capacity of 1300 mAh·g^−1^, ~90% capacity retention after 200 cycles, and high Coulombic efficiency, confirming the beneficial role of hierarchical porosity and carbon confinement in achieving both high capacity and long-term cycling stability. These results highlight the synergistic effect of high silicon loading, hierarchical porosity, and conductive carbon matrix on both capacity and cycling stability.

Graphene-wrapped Si nanoparticle composites (SiNP@G) have also proven effective, providing 1205 mAh·g^−1^ over 150 cycles [30]. Furthermore, yolk–shell carbon coatings created by chemical vapor deposition around Si nanoparticles offer internal void space to buffer lithiation-induced expansion, substantially improving structural reliability and cycling stability [31].

#### 2.1.2. Core–Shell and Metal Oxide Reinforced 0D Silicon Structures

Fang and co-workers [32] introduced a Si@TiO_2_ core–shell structure in which Si nanoparticles are encapsulated within a TiO_2_ shell containing internal void space. This design effectively buffers the large volume changes of silicon during cycling and delivered a reversible capacity of 804 mAh·g^−1^ at 0.1 C after 100 cycles, demonstrating the advantages of core–shell buffering layers for Si stabilization, highlighting the stabilizing role of oxide-based core–shell configurations.

Building on this concept, Park and colleagues [33] further enhanced structural and interfacial stability by mechanically assembling a Si/Ti_2_O_3_/rGO ternary nanocomposite. In this system, Ti_2_O_3_ acts as a mechanically robust and electrically conductive buffering phase, while reduced graphene oxide forms a flexible conductive network that improves electron transport and maintains structural integrity. As a result, the composite exhibited a reversible capacity of 950 mAh·g^−1^ after 100 cycles at a current density of 100 mA·g^−1^.

**Figure 3:**
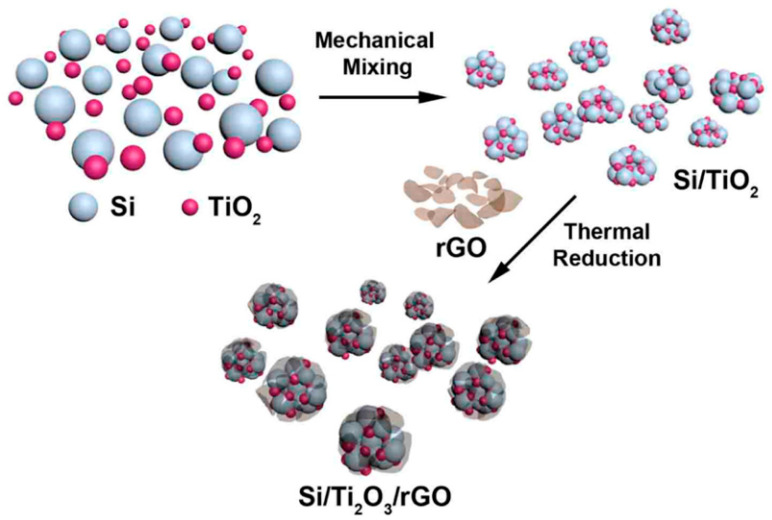
Schematic representation of the stepwise fabrication of the Si/Ti_2_O_3_/rGO ternary nanocomposites, illustrating the mechanical assembly and interfacial integration of silicon nanoparticles, Ti_2_O_3_, and reduced graphene oxide. Reprinted from Ref. [33], Copyright 2015, with permission from American Chemical Society.

The overall fabrication strategy of the Si/Ti_2_O_3_/rGO nanocomposites is schematically illustrated in Figure 3. As shown in Figure 3, the stepwise assembly enables intimate interfacial integration among silicon nanoparticles, the Ti_2_O_3_ buffering phase, and the conductive graphene network, which is critical for mitigating mechanical stress, maintaining electrical continuity, and facilitating efficient ion and electron transport during repeated cycling. Collectively, these studies underscore the continuous evolution of silicon nanoparticle engineering, where rational composite design at the nanoscale effectively mitigates mechanical stress, shortens ion and electron transport pathways, and substantially enhances electrochemical performance.

#### 2.1.3. Recycled and Waste-Derived Silicon Nanoparticles (Sustainable 0D Si)

Recent research has investigated the synthesis of zero-dimensional (0D) silicon nanoparticles from industrial by-products and recycled silicon sources, offering environmentally sustainable and cost-effective routes [34,35,36,37]. One notable example, reported by Liu and co-workers, utilizes fly ash, a coal combustion by-product, as a precursor to produce spherical Si NPs with an average diameter of approximately 50 nm [35]. Although fly ash is classified as hazardous waste due to its environmental and health hazards, it contains substantial amounts of silicon oxide, which can be transformed into high-purity crystalline silicon nanoparticles via acid leaching followed by magnesiothermic reduction.

The nanoparticles obtained through this approach exhibit a porous morphology, a high specific surface area of 220 m^2^·g^−1^, and a purity exceeding 99%. Electrochemical testing demonstrates that these 0D Si NPs achieve excellent lithium storage performance, retaining a high specific capacity of approximately 1030 mAh·g^−1^ after 500 cycles at a current density of 3.6 A·g^−1^, indicating both high stability and rate capability.

Another promising route for obtaining recycled silicon involves the recovery of high-purity silicon wafers from end-of-life photovoltaic (EoL-PV) modules. Eshraghi and colleagues demonstrated an effective recycling strategy for converting PV waste into nanoscale silicon suitable for high-performance lithium-ion battery anodes [34]. Their process employs sequential alkaline and acidic leaching steps to remove major metallic contaminants (Pb, Ag, Al), followed by high-energy ball milling to produce ultrahigh-purity nano-Si powders. The authors showed that the electrochemical behavior of the resulting silicon is strongly dependent on the leaching treatment: incomplete removal of Al and Ag residues suppresses effective particle size reduction during milling and leads to inferior cycling performance. Systematic variation in milling speed and duration revealed their pronounced influence on silicon crystallinity, particle-size distribution, and electrochemical reversibility. The recovered crystalline nano-Si exhibited particle sizes spanning the nano- to microscale and delivered a reversible capacity of ~1285 mAh·g^−1^ after 50 cycles.

**Figure 4:**
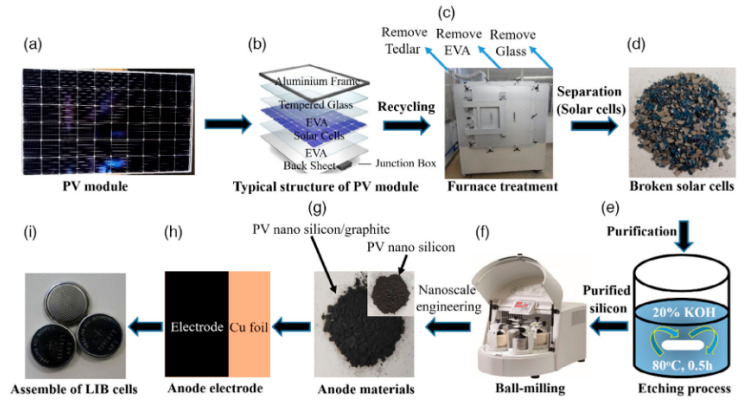
Schematic overview of the recycling and reuse pathway for silicon recovered from end-of-life photovoltaic modules: (**a**–**d**) disassembly and recovery of silicon cells; (**e**) chemical purification via KOH treatment; (**f**,**g**) conversion into nanosilicon and nanosilicon/graphite hybrid materials; and (**h**,**i**) fabrication of anode electrodes and assembly of lithium-ion battery cells. Ref. [36] is an open-access article distributed under the terms of the Creative Commons CC BY license, which permits unrestricted use, distribution, and reproduction in any medium provided the original work is properly cited.

Rahman and collaborators proposed an alternative PV-recycling strategy based on alkaline purification followed by mechanical size reduction, enabling the conversion of end-of-life photovoltaic modules into high-purity nanosilicon powders [36]. The overall process flow, from silicon cell recovery to anode fabrication and lithium-ion battery assembly, is schematically summarized in Figure 4. As illustrated in Figure 4, the recycling strategy integrates impurity removal, nanosilicon production, composite electrode fabrication, and cell assembly into a closed-loop pathway, highlighting the practical feasibility and scalability of converting end-of-life photovoltaic waste into functional lithium-ion battery anodes.

Through KOH-based impurity removal and subsequent ball milling, nano-Si with an average particle size of ~51 nm was obtained. When blended with graphite and evaluated as an anode material, the recycled nano-Si exhibited a reversible capacity of 426 mAh·g^−1^ after 600 cycles with ~70% capacity retention, a rate capability of 215 mAh·g^−1^ at 5 C, and a high average Coulombic efficiency of 99.4%, demonstrating the feasibility of waste-derived silicon for long-life lithium-ion batteries [36].

**Figure 5:**
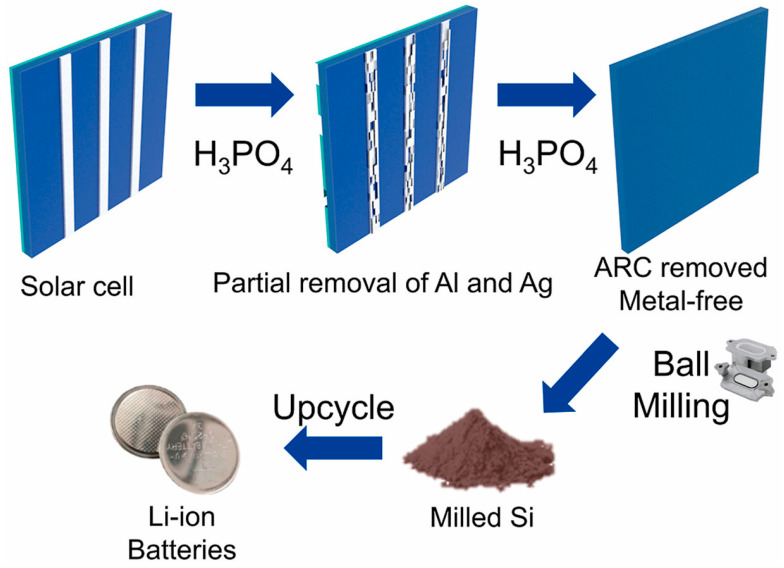
Schematic representation of the one-step upcycling strategy for expired silicon solar cells: phosphoric-acid-assisted removal of Al and Ag contacts and the SiN_x_ anti-reflective coating, generation of metal-free silicon, subsequent ball milling to obtain nanosilicon, and reuse of the recovered silicon as an active material for lithium-ion battery anodes. Reprinted from Ref. [37], Copyright 2023, with permission from Elsevier.

Similarly, Sim and co-workers introduced a simplified one-step recovery approach in which expired silicon cells are treated in hot diluted phosphoric acid [37]. This single-step process simultaneously removes the Al back-contact layer and the SiN_x_ anti-reflective coating while detaching Ag electrodes, achieving a silicon recovery yield of 98.9% with a purity of 99.2%. Subsequent ball milling reduces the particle size to approximately 100 nm. When evaluated as a lithium-ion battery anode, the recycled nano-Si delivers a specific capacity of 1086.6 mAh·g^−1^ after 500 cycles at 1C, while maintaining a Coulombic efficiency exceeding 99%. The overall upcycling pathway for end-of-life silicon solar cells is schematically illustrated in Figure 5. As shown in Figure 5, the phosphoric-acid-assisted treatment enables the simultaneous removal of metallic contacts and surface coatings in a single step, significantly simplifying the recycling process and reducing chemical and processing complexity. This streamlined pathway, followed by mechanical size reduction, directly supports the practical feasibility of producing metal-free nanosilicon from photovoltaic waste for reuse in lithium-ion battery anodes.

**Figure 6:**
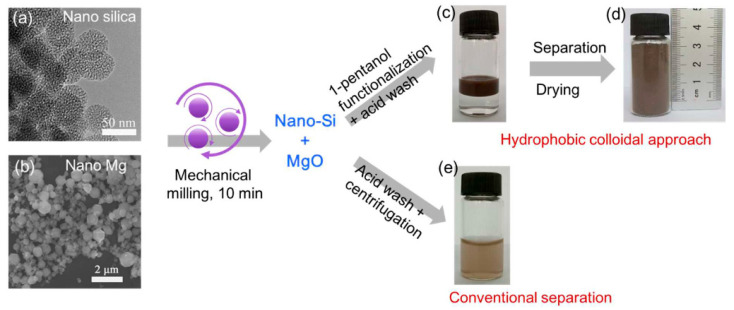
Formation pathway of hydrophobic colloidal silicon nanoparticles via nanoscale magnesiothermic reduction: (**a**) TEM image of nanosilica precursor and (**b**) SEM image of nano-Mg used for mechanical milling; (**c**) phase separation during acid washing, showing the hydrophobic nanosilicon layer floating above the aqueous MgCl_2_-containing solution; (**d**) dry nanosilicon powder obtained at gram-scale yield in a single batch; and (**e**) comparison with conventional acid washing and centrifugation, where significant loss of fine silicon particles leads to a turbid supernatant even after high-speed centrifugation. Reprinted from Ref. [38], Copyright 2017, with permission from American Chemical Society.

Other forms of zero-dimensional silicon have also emerged as strong candidates for high-performance lithium-ion battery anodes. Liu and colleagues from the Beijing National Laboratory for Molecular Sciences reported a nanoscale magnesiothermic reduction route for converting silica nanoparticles into colloidal Si NPs [38]. By using nanosized silica instead of bulk precursors, rapid and low-temperature conversion was achieved, with the reaction completed within only 10 min under room-temperature mechanical milling, in contrast to conventional high-temperature thermal processes. The resulting silicon nanoparticles possessed highly reactive surfaces and were subsequently modified with 1-pentanol to form a hydrophobic colloidal dispersion with an average particle size of ~40 nm, followed by a single-step carbon-coating treatment. The carbon-coated Si NPs exhibited excellent cycling stability, delivering a reversible capacity of 1756 mAh·g^−1^ after 500 cycles at a current density of 2.1 A·g^−1^. The overall fabrication pathway and the advantages of the hydrophobic separation strategy are schematically illustrated in Figure 6. As shown in Figure 6, surface-modification-induced phase separation enables the direct recovery of ultrasmall silicon nanoparticles without high-speed centrifugation, significantly reducing material loss and post-processing complexity while improving yield and scalability. This strategy demonstrates how rational surface engineering can simultaneously stabilize colloidal nanosilicon and enhance the practicality and industrial relevance of reduction-based synthesis routes.

From a sustainability and cost perspective, recycled silicon derived from industrial waste or end-of-life photovoltaic modules offers a promising alternative to electronic-grade silicon. Nevertheless, impurity control remains a critical issue, as residual metallic and dopant elements can catalyze parasitic reactions, destabilize the SEI, and reduce initial Coulombic efficiency. Balancing purification cost with electrochemical performance therefore represents a key challenge for the industrial adoption of recycled silicon anodes.

From a techno-economic standpoint, the reduced raw-material cost and utilization of existing waste streams constitute the primary advantages of waste-derived silicon routes. These benefits, however, are counterbalanced by the need for effective impurity control, as residual metallic species and dopants strongly influence particle size reduction, SEI stability, and initial Coulombic efficiency. Acid- or alkali-based purification steps thus represent a critical cost–performance trade-off: insufficient purification degrades electrochemical behavior, whereas excessive chemical treatment increases processing complexity and cost. Recent simplified strategies, such as one-step phosphoric-acid treatment and surface-modification-induced phase separation, demonstrate promising pathways for reducing purification overhead while maintaining high silicon purity and electrochemical performance. These developments indicate that the industrial viability of recycled silicon anodes will ultimately depend on achieving an optimal balance between purification efficiency, process simplicity, and electrode-level performance.

Taken together, these examples illustrate the broad diversity of synthetic strategies available for producing zero-dimensional silicon nanoparticles, underscoring the need for a comparative assessment of fabrication methods in terms of scalability, cost, and practical implementation.

#### 2.1.4. Comparative Analysis of 0D Silicon Nanoparticle Fabrication Methods

Among the available fabrication methods for 0D Si nanoparticles, ball milling remains one of the most industrially attractive approaches due to its scalability, low cost, and compatibility with diverse silicon feedstocks, including photovoltaic waste, metallurgical-grade silicon, and ferrosilicon. Through high-energy mechanical impact, bulk silicon can be reduced to particle sizes below ~150 nm, which helps alleviate pulverization by reducing absolute expansion strain during lithiation. Over-milling may induce undesirable agglomeration, but integration with carbon sources and partial amorphization can enhance structural stability and lithium-ion diffusivity [39,40].

Other approaches, including chemical etching, hydrothermal synthesis, magnesiothermic reduction, and CVD-based coatings, offer precise control over particle morphology and porosity but differ in scalability, cost, and practical challenges. A comparative overview of these fabrication methods is provided in Table 1.

Ball milling offers high throughput and low cost, making it the most feasible for industrial production, but requires strategies to control agglomeration, SEI formation, and initial Coulombic efficiency. Chemical etching and hydrothermal methods allow precise tuning of porosity and morphology but suffer from safety, scalability, and energy limitations, restricting commercial adoption. Magnesiothermic reduction and CVD-based coatings produce advanced architectures with excellent cycling performance, yet complexity, cost, and post-processing present significant barriers to mass production. Overall, balancing scalability, cost, safety, and electrochemical performance remains the primary commercialization challenge for 0D silicon nanoparticles.

These insights also highlight why research is shifting toward one-dimensional silicon architectures, where anisotropic geometries can mitigate some of the intrinsic limitations of 0D materials, enabling improved mechanical resilience and rate performance for next-generation lithium-ion battery anodes.

### 2.2. One-Dimensional (1D) Silicon Structures: Nanowires and Nanotubes

One-dimensional silicon architectures, particularly silicon nanowires (SiNWs) and silicon nanotubes (SiNTs), have attracted considerable attention as anode materials for lithium-ion batteries owing to their distinct structural advantages. First, their radial geometry effectively accommodates the large volume variations associated with lithiation and delithiation, thereby suppressing mechanical fracture and electrode pulverization. Second, the continuous one-dimensional framework enables efficient electron transport, which facilitates rapid charge transfer and improves electrochemical kinetics.

#### 2.2.1. One-Dimensional Silicon Nanowires

Among one-dimensional architectures, silicon nanowires have emerged as one of the most promising solutions for next-generation LIB anodes [39,40,41]. Their structural and electrochemical advantages make them suitable for high-power applications, faster charging, and extended driving range. Typically, SiNWs are grown directly on metallic current collectors, forming a mechanically robust and electrically continuous interface. This configuration offers several key benefits. First, the small diameter of the nanowires enables them to accommodate drastic volume fluctuations during lithiation and delithiation without mechanical fracture, which is a common failure mode for bulk or micron-sized silicon. Second, each nanowire remains in intimate electrical contact with the current collector, ensuring that the entire active material is electrochemically accessible. Third, the 1D geometry provides direct, continuous pathways for electron transport, unlike particle-based electrodes where electrons must traverse multiple interparticle boundaries with limited contact areas. Additionally, because every nanowire is anchored directly to the conductive substrate, the electrode can operate without polymer binders or conductive additives, reducing inactive mass and improving overall energy density. Extensive research has been devoted to the design and fabrication of 1D silicon-based anodes through diverse synthetic strategies.

**Figure 7:**
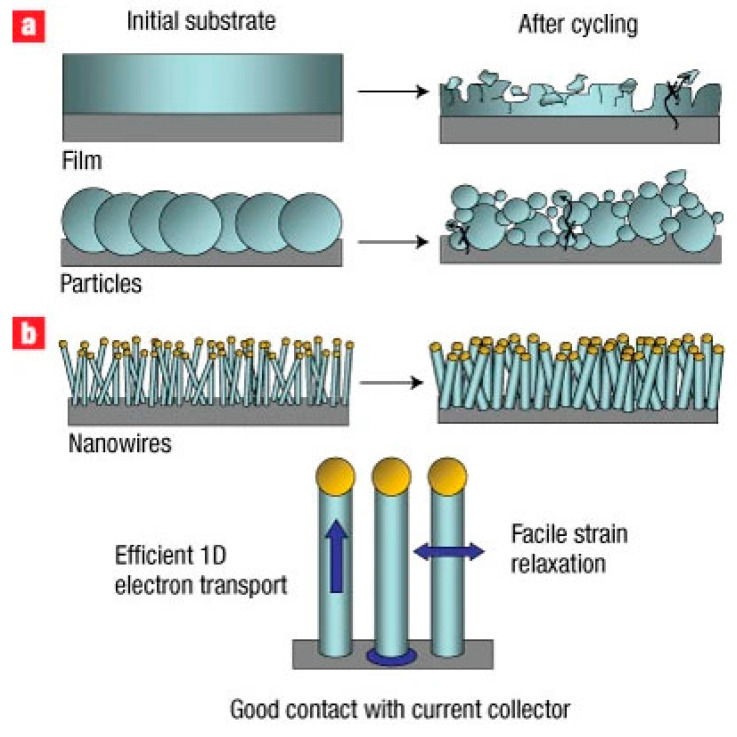
Schematic illustration of the morphological evolution of silicon anodes during electrochemical cycling and the corresponding impact of electrode architecture. (**a**) Conventional bulk silicon films and micron-sized particles undergo severe volumetric expansion (up to ~400%) upon lithiation, leading to mechanical pulverization, loss of electrical contact with the current collector, and rapid capacity fading. (**b**) One-dimensional silicon nanowire (Si NW) architectures grown directly on the current collector effectively accommodate large volume changes through radial and axial strain relaxation, preserving structural integrity and continuous electrical pathways. This design enables efficient one-dimensional electron transport along the nanowires and significantly improves cycling stability and rate performance. Reprinted from Ref. [42], Copyright 2008, with permission from Springer Nature.

To illustrate the fundamental mechanical advantages of one-dimensional silicon architectures, Figure 7 schematically compares the lithiation-induced morphological evolution of conventional bulk silicon electrodes and silicon nanowire–based anodes. As shown in Figure 7, the radial and axial strain relaxation enabled by the nanowire geometry suppresses pulverization and loss of electrical contact, directly explaining the superior cycling stability and rate performance observed for SiNW-based anodes.

In this context, Chan and co-workers [42] reported the preparation of silicon nanowires with diameters of approximately 90 nm via chemical vapor deposition in 2008. These SiNWs exhibited an initial Coulombic efficiency of 73% along with enhanced cycling durability, which was attributed to their ability to accommodate large volume changes without structural collapse.

Chen’s research team [43] reported the development of an innovative self-standing electrode architecture that combines high areal mass loading with long-term cycling stability. Their design employed carbon-coated silicon nanowires grown directly on highly conductive and mechanically flexible carbon fabric substrates via a nickel-catalyzed, single-step atmospheric chemical vapor deposition process. Owing to the uniform carbon coating and robust interfacial contact, the resulting SiNW-based electrode delivered an exceptionally high reversible capacity of approximately 3500 mAh·g^−1^ at a current density of 100 mA·g^−1^.

Keller and co-workers [44] produced Si NPs and SiNWs of various dimensions using laser pyrolysis and gold-catalyzed vapor–liquid–solid (VLS) growth, respectively, and systematically compared their electrochemical behavior as LIB anode materials. Their study demonstrated that particle morphology plays a decisive role: although Si NPs exhibited relatively high specific capacity, they suffered from poor cycling stability. The initial coulombic efficiency scaled linearly with specific surface area, and larger Si NPs tended to form crystalline Li_15_Si_4_ and undergo electrochemical sintering, accelerating capacity fading.

In contrast, SiNWs, particularly the smallest SiNW9 sample, displayed lower intrinsic capacity but outstanding coulombic efficiency and capacity retention. The advantages stem from their 1D architecture, which helps preserve a porous three-dimensional electrode network, facilitates lithium diffusion, and prevents sintering during repetitive cycling. A further practical benefit is that SiNWs can be grown directly on metallic substrates, forming binder-free, electrically continuous interfaces with current collectors [45,46]. Their linear geometry also provides rapid electron pathways and accommodates substantial volume fluctuations without structural pulverization [47,48]. The primary drawback parallels that of other nanoscale materials: a high surface-to-volume ratio results in large initial irreversible capacity.

Prosini and collaborators [49] synthesized SiNWs with diameters of 200–500 nm via gold-catalyzed chemical vapor deposition. When tested as LIB anodes, these SiNWs delivered an initial discharge capacity of ~2.15 mAh at 0.05 mA and demonstrated stable cycling. Although the first cycle showed an irreversible loss of ~0.3 mAh, increasing the current density led to reduced capacity degradation. In a full-cell configuration paired with LiFePO_4_ at 0.12 mA, the SiNW anode delivered an initial discharge capacity of 0.8 mAh, gradually declining to 0.6 mAh after 10 cycles, while coulombic efficiency increased to 95%. The authors attributed the capacity decay to mismatched electrode balancing, leading to lithium consumption in the cathode exceeding reinsertion into the anode.

Yu and co-authors [50] advanced this field by growing SiNWs in situ on graphite via molten-salt electrolysis, yielding a SiNWs/G@C composite. At a current rate of 0.5 C, the composite exhibited an initial discharge capacity of ~674 mAh·g^−1^ and maintained 90.04% of this capacity after 100 cycles. The exceptional stability was attributed to the formation of robust SiC bonding between the SiNWs and graphite, which enhanced mechanical integrity and interfacial adhesion. By tuning the electrolysis conditions, the SiC content could be controlled, enabling further optimization of cycling stability.

Sun and co-workers [51] fabricated ultra-thin SiNWs (UT-SiNWs) with average diameters of 30 nm and 10 nm using a bimetal-assisted chemical etching method. The 30 nm wires exhibited superior stability compared to conventional 100 nm SiNWs, delivering a discharge capacity of 1066 mAh·g^−1^ at 300 mA·g^−1^ and retaining 87.5% of their capacity after 50 cycles. Their enhanced performance was attributed to the smaller diameter and increased SiO_x_ content, which promoted the formation of a mechanically stable SEI and suppressed parasitic interfacial reactions.

Lu and collaborators proposed an innovative recycling approach that converts photovoltaic waste silicon into high-performance Si nanowire architectures anchored on reduced graphene oxide (SiNWs@rGO) for LIB applications [52]. The method consists of two main steps. First, waste-derived Si particles are assembled with graphene oxide sheets, forming a layered structure in which Si particles are confined between GO layers. Next, an electrothermal shock treatment is applied, rapidly heating and cooling the composite (≈2100 K for ~10 ms). This ultrafast thermal process induces the growth of ∼50 nm Si nanowires within the confined interlayer spaces of the graphene oxide matrix. The restricted geometry provided by rGO and the natural oxide shell on the Si particles play crucial roles in directing nanowire formation. When used as a binder-free LIB anode, the resulting SiNWs@rGO electrode demonstrates a Coulombic efficiency of 89.5% and outstanding long-cycle stability, maintaining 2381.7 mAh·g^−1^ at 1 A·g^−1^ for over 500 cycles at a silicon content of 76%.

Another promising synthesis route for 1D silicon nanowires is molten salt electrolysis, which offers an inexpensive and environmentally benign alternative. In this method, SiO_2_ is electrochemically reduced in a molten salt medium, typically CaCl_2_, at elevated temperatures. Upon applying an external voltage, Si^4+^ ions are reduced to elemental silicon, producing nanowire structures directly on a conductive substrate. This technique stands out for its simplicity, scalability, and reliance on abundant, non-toxic starting materials, avoiding hazardous gaseous precursors such as silane used in vapor-based processes. While issues such as nanowire uniformity, crystallinity control, and high-temperature operation remain challenges, molten salt electrolysis represents a highly promising green pathway for producing SiNWs for advanced lithium-ion batteries [39,40].

#### 2.2.2. One-Dimensional Silicon Nanotubes

Silicon nanotubes (SiNTs) represent a distinctive class of one-dimensional silicon architectures characterized by their hollow tubular morphology and nanoscale radial dimensions. This engineered void space plays a crucial role in buffering the substantial volume fluctuations that accompany lithiation and delithiation. As the silicon expands during cycling, the internal cavity provides mechanical accommodation, thereby reducing fracture risks and preserving structural integrity [53].

Wen and co-authors [54] developed SiNTs via a two-step process in which rod-like Ni–N_2_H_4_ structures served as templates to first produce silica nanotubes, which were subsequently converted to silicon nanotubes through magnesiothermic reduction. The resulting SiNTs exhibited diameters near 15 nm, lengths of 50–200 nm, good crystallinity, and well-defined porosity. When evaluated as LIB anodes, the SiNTs outperformed commercial Si-325 mesh. Although both electrodes displayed initial capacity loss due to SEI formation, irreversible Li uptake, and material pulverization, the Si-325 mesh suffered far more pronounced degradation. After approximately six cycles, the SiNT electrodes stabilized, and by the tenth cycle their discharge capacity was roughly double that of the Si-325 mesh. Their superior rate performance and retention over 90 cycles were attributed to the nanotube morphology, increased electrolyte contact, and a stabilized interface, despite some capacity limitations likely stemming from incomplete silica reduction.

Zhao and co-workers [55] developed a novel one-dimensional tubular silicon/nitrogen-doped carbon composite (Si@NC) featuring a core–shell configuration, using a silicon–magnesium alloy and polydopamine as both template and carbon/nitrogen precursor. Scanning electron microscopy (SEM) analysis revealed uniformly distributed silicon domains with an average diameter of ~100 nm. Benefiting from the synergistic effects of the tubular architecture and nitrogen-doped carbon confinement, the Si@NC composite delivered a high reversible capacity and fast redox kinetics, maintaining a stable capacity of 583.6 mAh·g^−1^ at 0.5 A·g^−1^ over 200 cycles. The engineered nanotube framework effectively buffers volume expansion while the conductive N-doped carbon shell enhances structural integrity and electrochemical stability.

Park et al. [56] fabricated carbon-coated SiNTs and demonstrated remarkable electrochemical performance in both half-cell and full-cell configurations. In half-cells, the SiNTs delivered an initial discharge capacity of 3648 mAh·g^−1^ and a charge capacity of 3247 mAh·g^−1^ at 0.2C, corresponding to a coulombic efficiency of 89%. Even at an ultrahigh rate of 5C (15 A·g^−1^), the electrodes maintained a charge capacity of 2878 mAh·g^−1^. Full cells assembled with SiNT anodes and LiCoO_2_ cathodes delivered initial capacities exceeding 3000 mAh·g^−1^ at 3C and 5C. After 200 cycles at 1C, the SiNT-based full cell retained 89% of its capacity, vastly outperforming conventional graphite anodes. This strong rate capability and cycling durability stem from the interconnected porous SiNT array, which provides abundant active surface area and accommodates large volume swings during alloying/dealloying, enabling significant pseudocapacitive Li storage.

Song and co-workers [46] successfully constructed ordered Si nanotube arrays on stainless-steel substrates through a synthesis strategy combining hard templating with chemical vapor deposition. The presence of internal hollow channels and inter-tube void spaces effectively accommodated volume expansion during cycling, enabling the electrode to retain a capacity of about 2500 mAh·g^−1^ after 50 cycles.

**Figure 8:**
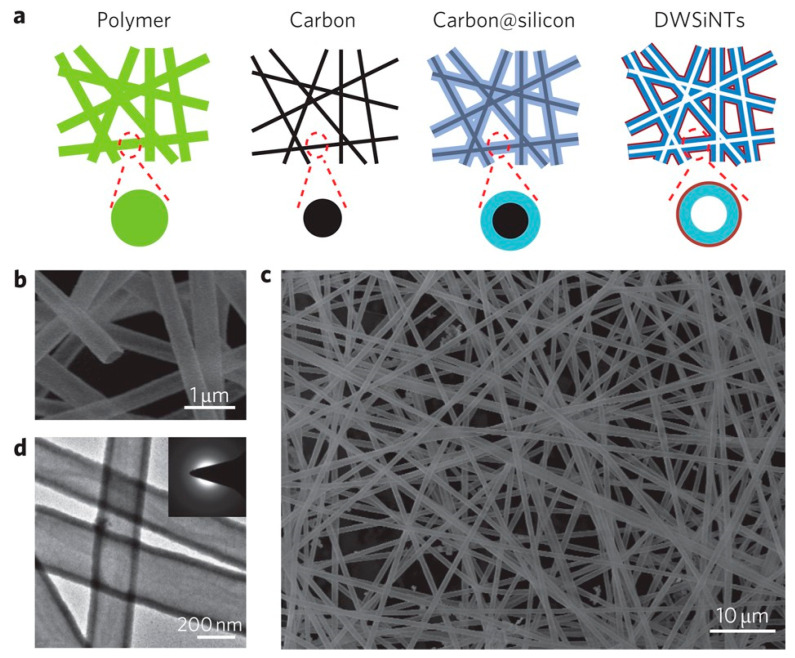
Fabrication and structural characterization of double-walled Si/SiO_x_ nanotubes (DW-Si/SiO_x_ NTs): (**a**) Schematic illustration of the synthesis route: electrospun polymer nanofibers (green) are carbonized, coated with silicon (blue) via CVD, and finally selectively oxidized at 500 °C to remove the inner carbon template (black), leaving a continuous silicon tube constrained by an outer SiO_x_ layer (red); SEM images at low (**b**) and high (**c**) magnification, highlighting the uniform, interconnected nanotube network; TEM image (**d**) showing the hollow dual-layer structure with smooth tube walls. Reprinted from Ref. [57], Copyright 2012, with permission from Springer Nature.

Furthermore, Wu and co-workers [57] proposed a unique double-walled silicon–silicon oxide nanotube (DW-Si/SiO_x_ NT) architecture, where the inner silicon serves as the active material and the outer SiO_x_ shell provides mechanical and chemical protection. The schematic and microscopy images in Figure 8 highlight the formation of hollow nanotubes with uniform morphology and dual-layer confinement. Electrochemical testing revealed that this design preserved ~94% of the initial capacity after 500 cycles, demonstrating how structural engineering at the nanoscale effectively mitigates silicon volume expansion and stabilizes the electrode–electrolyte interface.

Beyond mechanical buffering, the hollow tubular geometry of silicon nanotubes inherently provides a high specific surface area and shortened lithium-ion diffusion paths. These features enhance electrolyte accessibility, reduce polarization, and support improved rate capability, complementing the strain-accommodation mechanism illustrated in Figure 8.

Despite these merits, the practical implementation of SiNT-based anodes remains closely linked to the complexity of their synthesis routes, motivating further comparative evaluation of fabrication strategies. While silicon nanotubes and nanowires clearly demonstrate the mechanical and electrochemical advantages of one-dimensional architectures, their broader applicability is ultimately governed by the scalability, cost, and practical constraints of the fabrication methods employed.

#### 2.2.3. Comparative Analysis of 1D Silicon Nanostructure Fabrication Methods

One-dimensional silicon nanostructures, including silicon nanowires and silicon nanotubes, can be synthesized via diverse methods, each offering unique control over morphology, crystallinity, and porosity, but differing significantly in scalability, cost, and practical implementation challenges. Key fabrication strategies include vapor–liquid–solid (VLS) growth, chemical vapor deposition (CVD), molten salt electrolysis, template-assisted or hard-templating methods, and electrochemical or chemical etching. A comparative overview of these approaches is provided in Table 2.

SiNWs generally provide continuous electron-conduction pathways, excellent mechanical resilience, and high Coulombic efficiency. Their radial geometry allows for effective accommodation of volume changes, minimizing fracture and capacity fading. However, the most widely used fabrication routes, such as VLS or CVD, require high temperatures, metal catalysts, and precise control over nanowire dimensions. These factors increase production cost, reduce scalability, and limit near-term industrial feasibility. Template-assisted methods can yield highly uniform and hollow SiNW structures, further improving lithium-ion transport and stress accommodation, yet the multistep processes, template removal, and reproducibility challenges hinder large-scale adoption. Molten salt electrolysis has recently emerged as a promising, environmentally benign alternative. This approach enables direct growth of SiNWs on conductive substrates using inexpensive precursors and avoids hazardous gaseous reagents. While this method shows potential for scale-up, challenges remain in achieving uniformity, controlling crystallinity, and producing nanowires with consistent diameters.

SiNTs, by contrast, offer hollow tubular morphologies that effectively buffer the substantial volume expansion during lithiation and delithiation. The internal cavity, combined with a high specific surface area, facilitates ion diffusion and enhances rate performance. Nevertheless, their fabrication often relies on templating strategies or specialized equipment, making large-scale production technically demanding and costly. Despite these advantages, both SiNWs and SiNTs share common limitations associated with one-dimensional nanostructures: relatively low volumetric energy density, high surface reactivity leading to SEI formation, and limited compatibility with conventional roll-to-roll electrode manufacturing.

In summary, one-dimensional silicon architectures outperform zero-dimensional nanoparticles in terms of mechanical robustness, electron transport, and cycling stability, yet each fabrication method presents trade-offs between performance, cost, and scalability. While VLS/CVD and templating routes offer precise structural control, they remain expensive and low-throughput. Molten salt electrolysis presents a more scalable and environmentally friendly pathway, but further optimization is required to ensure uniformity and reproducibility. Understanding these trade-offs is critical for bridging laboratory-scale innovation with industrial implementation.

Building on these insights, two-dimensional silicon-based anode materials naturally emerge as the next step. Their planar architectures offer additional opportunities to enhance mechanical integrity, ion transport, and interfacial stability, potentially overcoming some of the scalability and volumetric energy density limitations observed for 1D silicon structures.

### 2.3. Two-Dimensional (2D) Silicon Structures: Nanofilms and Nanosheets

Two-dimensional (2D) silicon-based materials have emerged as a compelling class of nanostructured anodes for lithium-ion batteries owing to their ultrathin thickness and extended lateral dimensions. Such architectures offer shortened lithium-ion diffusion pathways, enlarged electrode–electrolyte interfacial areas, and accelerated interfacial charge-transfer kinetics, collectively contributing to enhanced electrochemical performance [39,40]. By effectively decoupling ion transport from bulk diffusion limitations, well-engineered 2D silicon anodes can deliver improved rate capability and cycling stability.

#### 2.3.1. Two-Dimensional Silicon Nanofilms

Two-dimensional (2D) silicon nanofilms are characterized by an ultrathin thickness, typically ranging from several nanometers to a few tens of nanometers, while extending laterally in a planar geometry. In these structures, silicon atoms are arranged within a continuous two-dimensional framework, offering distinct electrochemical advantages when employed as anode materials in lithium-ion batteries. Owing to their reduced thickness, 2D Si nanofilms enable shortened lithium-ion diffusion paths, rapid interfacial charge transfer, and intimate contact with the electrolyte.

At present, 2D silicon nanofilms are predominantly fabricated using two vapor-phase techniques. Chemical vapor deposition (CVD) relies on the thermal decomposition of gaseous silicon precursors in the presence of catalysts, leading to conformal silicon film growth on the substrate surface. Alternatively, physical vapor deposition (PVD) methods, including evaporation and sputtering, vaporize solid silicon sources and subsequently deposit them as thin films onto substrates. Both approaches allow precise control over film thickness and microstructure, which are critical for optimizing electrochemical behavior [58,59,60].

Suresh and co-workers [61] employed a methanol-mediated floating catalyst chemical vapor deposition (FCCVD) strategy to deposit a silicon nanofilm directly onto a macroscopic carbon nanotube network (CNM) current collector, followed by the introduction of a single-layer graphene capping film. This graphene-covered silicon nanofilm effectively suppressed interfacial delamination and structural degradation during cycling. As a result, the electrode exhibited outstanding cycling durability exceeding 1000 charge–discharge cycles, with an average gravimetric capacity of 806 mAh·g^−1^. Notably, the volumetric capacity remained as high as 2821 mAh·cm^−3^ even after prolonged cycling, highlighting the robustness of this layered architecture.

Graetz and colleagues [62] demonstrated that a 100 nm thick amorphous silicon thin film could deliver a stable specific capacity of approximately 2000 mAh·g^−1^ over 50 cycles, highlighting the beneficial role of nanoscale thickness in maintaining electrochemical reversibility. Similarly, Chen and co-workers [63] deposited a 275 nm thick amorphous Si layer onto a copper substrate and reported a reversible capacity of 2200 mAh·g^−1^ after 500 cycles, underscoring the excellent cycling stability achievable with well-adhered thin-film electrodes. Placke’s research team [64] further expanded this concept by fabricating Mg–Si thin films via magnetron sputtering, in which silicon was embedded within an electrochemically active magnesium silicate matrix. This composite thin-film structure exhibited a reversible capacity of approximately 1000 mAh·g^−1^ and retained 96% of its capacity after 400 cycles, indicating enhanced structural robustness imparted by the matrix-assisted design. In another approach, Sun’s research team [65] employed electrodeposition to fabricate silicon films on a three-dimensional nickel foam substrate. The resulting electrode delivered an exceptionally high reversible capacity exceeding 2800 mAh·g^−1^ at a current density of 360 mA·g^−1^ over 80 cycles, benefiting from both the thin-film configuration and the porous conductive scaffold.

Among various 2D silicon allotropes, silicene, an atomically thin graphene-like form of silicon, has attracted growing interest due to its unique electronic structure and high theoretical capacity. Nevertheless, the practical implementation of silicene remains challenging because of its intrinsic chemical reactivity and poor stability under ambient conditions. To address these limitations, recent studies have demonstrated a scalable exfoliation strategy in which end-of-life (EoL) photovoltaic (PV) panel-derived silicon powder is converted into multilayer silicene with thicknesses ranging from approximately 1.7 to 3 nm [66]. When integrated with graphite to form a composite anode, the resulting silicene-based electrode exhibits a Coulombic efficiency exceeding 97% and maintains a stable reversible capacity of ~290 mAh·g^−1^ at 1 C over 500 cycles, highlighting the feasibility of recycled silicon sources for advanced 2D anode materials.

From a structural perspective, the planar morphology of silicon thin films plays a decisive role in their electrochemical behavior. The large surface-area-to-volume ratio reduces lithium-ion diffusion distances and facilitates rapid electron transport, thereby minimizing polarization and improving rate capability. Moreover, the continuous 2D geometry promotes uniform stress distribution during volume changes associated with alloying and dealloying reactions, effectively mitigating mechanical degradation. These features collectively render silicon thin films a compelling platform for the development of durable and high-performance 2D silicon-based anodes.

#### 2.3.2. Two-Dimensional Silicon Nanosheets

Beyond silicene, silicon nanosheets (Si NSs) represent another important subclass of 2D silicon structures and are typically fabricated through exfoliation, reduction, or vapor-phase growth approaches. In addition to continuous nanofilms, silicon nanosheets (Si NSs) represent another important class of 2D silicon anode materials. Compared with other silicon nanostructures, Si NSs provide exceptionally short lithium-ion transport distances perpendicular to the sheet plane, which significantly enhances ion diffusion kinetics and interfacial charge transfer [67]. Moreover, silicon nanosheets can be synthesized through a broader range of processing routes, including chemical etching and exfoliation, vapor-phase deposition, and template-assisted methods.

For instance, Chen and co-workers developed a sheet-stacked porous silicon/carbon (Si/C) composite via magnesiothermic reduction followed by CO_2_-assisted carbon coating, employing silicon waste generated during diamond-wire cutting in PV manufacturing as the starting material [68,69]. The resulting Si/C anode delivered a reversible capacity of 693 mAh·g^−1^ after 300 cycles at a current density of 1.0 A·g^−1^, demonstrating both effective waste reutilization and long-term cycling stability.

**Figure 9:**
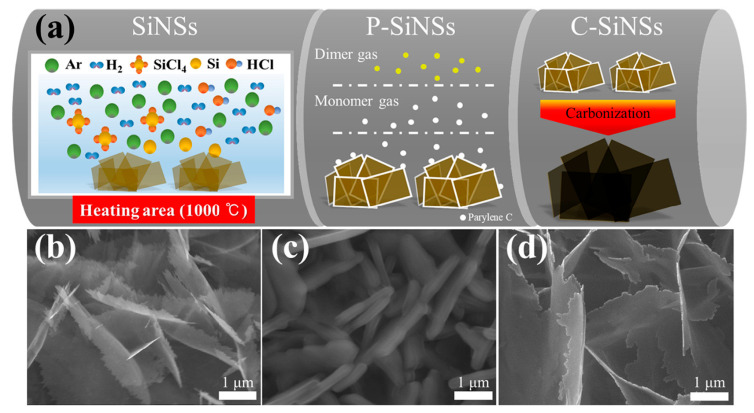
Synthesis and structural characterization of carbon-coated silicon nanosheets (C-SiNSs): (**a**) Schematic illustration of the fabrication process: Si nanosheets (SiNSs) are grown on graphite foil, coated with parylene C (P-SiNSs), and subsequently pyrolyzed at 950 °C in Ar to yield uniform carbon-coated SiNSs (C-SiNSs); (**b**–**d**) SEM images showing pristine SiNSs, parylene C–coated SiNSs, and final C-SiNSs, respectively, highlighting the reduction in thickness after carbonization and the uniform coating morphology. Reproduced from Ref. [70], Copyright 2021, with permission from Elsevier.

Surface engineering plays a pivotal role in enhancing the electrochemical stability of two-dimensional silicon architectures. As illustrated in Figure 9, Park and colleagues [70] synthesized uniformly carbon-coated Si nanosheets through a three-step process involving CVD growth, parylene coating, and thermal carbonization. Figure 9 demonstrates that precise control over carbon-shell thickness enables simultaneous enhancement of electronic conductivity and effective buffering of silicon volume expansion, directly accounting for the nearly 100% Coulombic efficiency and stable capacity retention observed during long-term cycling. Among the investigated configurations, a 10 nm carbon coating yielded the most balanced performance, achieving nearly 100% Coulombic efficiency and a high reversible capacity of approximately 2100 mAh·g^−1^ after 300 cycles.

To further enhance the electrochemical performance of two-dimensional silicon nanosheets as anode materials for lithium-ion batteries, Tang and co-workers developed porous Si NSs with a thickness of approximately 30 nm using a soft-templating strategy followed by magnesiothermic reduction [71]. This approach involves the initial synthesis of mesoporous SiO_2_ nanosheets and their subsequent conversion into silicon through magnesiothermic reduction, with NaCl employed as a heat scavenger to suppress structural collapse. High-resolution transmission electron microscopy (HRTEM) revealed that the resulting Si NSs consist of interconnected silicon nanocrystals with an average size of ~10 nm, forming a robust porous network. When evaluated as LIB anodes, these porous Si NSs exhibited outstanding rate capability and cycling stability, delivering a reversible capacity of approximately 800 mAh·g^−1^ after 900 cycles at an ultrahigh current density of 8400 mA·g^−1^.

Yao and co-workers [72] reported a two-dimensional porous sandwich-like silicon/carbon nanosheet architecture, in which porous silicon nanomembranes were grown symmetrically on both sides of reduced graphene oxide (rGO) sheets, followed by conformal carbon coating, forming a C/Si–rGO–Si/C structure. The coexistence of micropores and mesopores within this composite facilitated rapid lithium-ion diffusion, while the porous silicon layers provided efficient electron transport pathways. Simultaneously, the outer carbon coating enhanced electrical conductivity and promoted the formation of a stable solid electrolyte interphase. As a consequence, the C/Si–rGO–Si/C electrode delivered a high reversible capacity of 1187 mAh·g^−1^ after 200 cycles at 0.2 A·g^−1^ and maintained 894 mAh·g^−1^ after 1000 cycles at 1 A·g^−1^. Even under high-rate conditions, the electrode exhibited excellent performance, retaining capacities of 694 mAh·g^−1^ at 5 A·g^−1^ and 447 mAh·g^−1^ at 10 A·g^−1^, underscoring the effectiveness of rational 2D structural design for silicon-based anodes.

Beyond planar 2D architectures, hybrid electrode designs that integrate low-dimensional silicon nanostructures with three-dimensional current collectors have also demonstrated significant promise. Recently, Saana Amiinu and collaborators [73] reported a binder-free, high-capacity anode composed of indium-seeded silicon nanowires grown on a three-dimensional copper–silicide (Si NWs@3D-CS) current collector. In this method, a 3D nanostructured copper–silicide network is first fabricated by functionalizing a copper foil surface with silylbenzene, followed by the introduction of indium catalyst seeds that act as uniform nucleation sites. Subsequent thermal decomposition of silicon precursors in a liquid medium induces the growth of a well-interconnected Si nanowire network. Owing to its highly branched architecture, with an average branch size of ~140 nm, the 3D copper–silicide scaffold provides a large specific surface area that enables homogeneous catalyst distribution and the formation of Si nanowires with an average diameter of ~70 nm. As a result, the Si NWs@3D-CS electrode exhibits exceptional electrochemical performance, including a Coulombic efficiency exceeding 99.6%, stable operation over more than 900 cycles with 88.7% capacity retention, and a high volumetric capacity of approximately 1086.1 mAh·cm^−3^ at a rate of 5C. These results underscore the effectiveness of combining low-dimensional silicon nanostructures with architected current collectors to achieve high stability, high efficiency, and practical volumetric energy density.

While such hybrid designs demonstrate how two-dimensional silicon architectures can be effectively integrated with three-dimensional current collectors to achieve outstanding electrochemical performance, their broader applicability is strongly governed by fabrication strategy, structural scalability, and electrode-level trade-offs.

#### 2.3.3. Comparative Analysis of 2D Silicon Nanostructure Fabrication Methods

Two-dimensional silicon nanostructures represent a significant conceptual advance in the development of high-performance silicon anodes, as their planar geometry fundamentally alters stress distribution, ion transport, and interfacial stability during repeated lithiation and delithiation. Unlike particulate or filamentary architectures, 2D silicon materials confine volumetric expansion primarily within the plane of the electrode, effectively suppressing crack initiation and catastrophic mechanical failure. These intrinsic geometric advantages underpin the excellent cycling stability frequently reported for silicon nanofilms and nanosheets.

From a fabrication perspective, vapor-phase deposition techniques, such as chemical vapor deposition, floating-catalyst CVD, and magnetron sputtering, offer unparalleled control over film thickness, uniformity, and adhesion to current collectors. This level of structural precision enables homogeneous current distribution, uniform solid electrolyte interphase formation, and stable long-term electrochemical performance. However, these advantages come at the cost of high processing temperatures, vacuum-based equipment, low deposition rates, and significant capital investment. As a result, vapor-deposited 2D silicon electrodes are typically produced at extremely low mass loadings, which severely limits their achievable areal capacity and restricts their relevance to model systems rather than scalable battery technologies.

In contrast, solid-state and solution-derived fabrication routes, including magnesiothermic reduction, chemical etching, and exfoliation-based synthesis, enable the formation of silicon nanosheets with higher material loading and improved scalability. These approaches facilitate the construction of porous or layered 2D architectures that enhance electrolyte penetration, shorten lithium-ion diffusion pathways, and buffer mechanical stress through internal voids or composite interfaces. Nevertheless, such methods often involve multistep processing, residual oxide phases, and challenges in controlling nanosheet thickness and lateral uniformity. These factors introduce variability in electrochemical performance and complicate reproducibility at larger scales.

Electrodeposition-based methods occupy an intermediate position between vapor-phase and solid-state routes. They offer moderate scalability and cost advantages while allowing direct integration of silicon layers onto conductive substrates with tunable thickness. However, maintaining defect-free coverage, strong adhesion, and uniform current distribution over large electrode areas remains nontrivial, particularly at higher areal loadings. Consequently, electrodeposited 2D silicon films have yet to demonstrate consistent performance under commercially relevant conditions.

Despite their structural and electrochemical advantages, two-dimensional silicon architectures share several intrinsic limitations that constrain their practical implementation. The same planar confinement that stabilizes mechanical behavior also limits the amount of active material that can be incorporated without compromising electrode integrity. Furthermore, many 2D silicon fabrication routes remain poorly compatible with conventional roll-to-roll electrode manufacturing, especially when binder-free or substrate-grown configurations are employed. These incompatibilities pose significant barriers to large-scale adoption within existing lithium-ion battery production lines.

Overall, two-dimensional silicon nanostructures provide critical insights into stress confinement, interfacial engineering, and charge-transport optimization in silicon-based anodes. However, their commercialization will require further advances in scalable fabrication, increased areal capacity, and hybrid electrode designs that integrate the mechanical advantages of planar architectures with the material loading demands of practical batteries. These considerations naturally motivate the transition toward three-dimensional silicon architectures, where hierarchical structuring offers greater flexibility in balancing stability, scalability, and volumetric energy density in next-generation lithium-ion batteries.

### 2.4. Three-Dimensional (3D) Porous Silicon Structures

Compared with the previously discussed silicon nanostructures, three-dimensional porous silicon exhibits a highly developed pore network that effectively provides internal buffering space to accommodate the substantial volume variation in Si during lithiation and delithiation. In addition, its large specific surface area facilitates lithium-ion transport and diffusion, thereby improving both rate capability and long-term cycling stability. As the discussion progresses from two-dimensional to three-dimensional silicon architectures, porous silicon nanostructures emerge as a particularly effective strategy for enhancing the electrochemical performance of silicon anodes. The introduction of an interconnected pore network provides internal free volume that accommodates silicon expansion during lithiation, thereby minimizing macroscopic electrode swelling and preventing mechanical fracture. This structural buffering effect also promotes the formation of a more stable SEI, which in turn supports reliable electronic conductivity and long-term cycling stability of the anode [39,40,74].

Porous silicon is mainly synthesized through electrochemical etching [75,76,77], magnesiothermic reduction [78,79], templating strategies [80,81], or combinations of these methods.

#### 2.4.1. Porous Nanoparticles and Hollow Spheres

Lv and co-workers [82] prepared dopant-controllable porous silicon nanoparticles by combining ball milling with acid etching. Compared with intrinsic silicon, the resulting porous nanosilicon exhibited a high porosity of up to 45.8% and an electrical conductivity increase of nearly nine orders of magnitude. Electrochemical testing demonstrated that the porous Si anode delivered a stable capacity of approximately 2000 mAh·g^−1^ at 0.5 C over 100 cycles, maintained an excellent rate capability of 1600 mAh·g^−1^ at 5 C, and retained a reversible capacity exceeding 1500 mAh·g^−1^ at 1 C for up to 940 cycles.

Yao and collaborators [10] synthesized interconnected hollow silicon sub-microspheres using silica nanoparticles as sacrificial templates. The fabrication involved silicon-templated SiH_4_ chemical vapor deposition, followed by selective etching of the silica core to form hollow architectures. The resulting structure consisted of uniformly sized hollow spheres interconnected through shared shells, providing a mechanically robust framework and continuous pathways for electron and lithium-ion transport. When evaluated as a lithium-ion battery anode, the hollow-sphere silicon delivered a high initial discharge capacity of 2725 mAh·g^−1^. Over 700 charge–discharge cycles, the capacity decay remained below 8% per 100 cycles, while the Coulombic efficiency stabilized at approximately 99.5% in later cycles. The synergistic combination of hollow interiors and porous shells effectively mitigated silicon volume expansion and promoted rapid lithium-ion diffusion, resulting in outstanding electrochemical stability and performance that significantly surpassed conventional graphite anodes.

**Figure 10:**
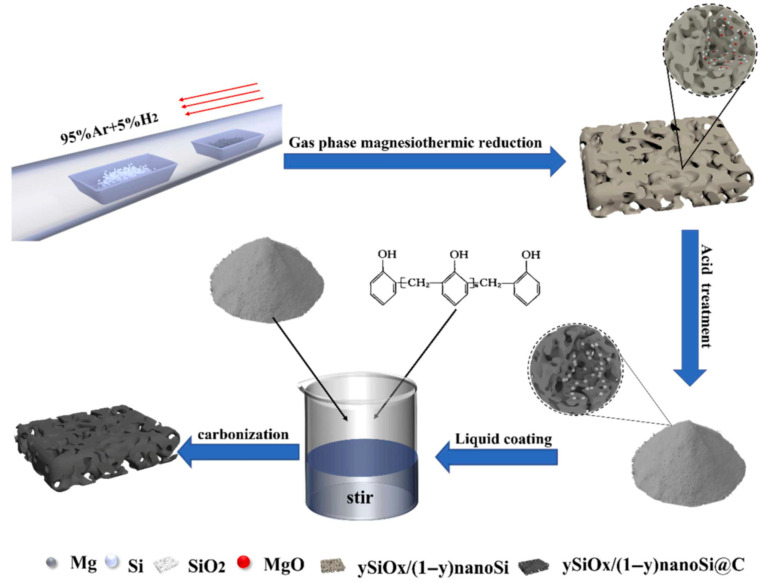
Fabrication scheme of hierarchical ySiO_x_/(1 − y)nanoSi@C composites. The schematic illustrates the stepwise synthesis of the composites: (i) mixing of silica with NaCl, (ii) magnesiothermic reduction with Mg powder under Ar/H_2_ atmosphere at 650 °C, (iii) acid washing to remove byproducts, and (iv) carbon coating of the resulting ySiO_x_/(1 − y)nanoSi powders to obtain the final hierarchical porous composite. Reproduced from Ref. [83], Copyright 2023, with permission from Elsevier.

Beyond hollow architectures, porous silicon–based composite structures represent another effective strategy to address the intrinsic challenges of Si anodes. Highly porous SiO_x_/nanoSi@C composites have been synthesized via gas-phase magnesiothermic reduction in low-cost fumed silica at 650 °C [83]. Magnesium vapor enables controlled penetration into the silica matrix, ensuring uniform formation of SiO_x_ and nano-Si domains while suppressing incomplete reduction. Figure 10 schematically illustrates the stepwise synthesis route, including magnesiothermic reduction and subsequent carbon coating, which together establish a hierarchical porous architecture with improved electrical connectivity.

When evaluated as an anode for lithium-ion batteries, the SiO_x_/nanoSi@C electrode maintains a reversible capacity of 1067.9 mAh·g^−1^ after 400 cycles at 1 A·g^−1^. This performance arises from the complementary functions of each component: nano-Si provides high capacity, the porous SiO_x_ matrix buffers volume expansion through the formation of Li_2_O and lithium silicates, and the carbon coating stabilizes the electrode–electrolyte interface while enhancing electronic transport [83].

More broadly, magnesiothermic reduction in silica has been extensively investigated as a versatile route for fabricating porous silicon structures [39,40]. In this process, magnesium acts as a strong reducing agent, converting silica into silicon while simultaneously generating a porous framework. Owing to its simplicity, use of inexpensive and abundant raw materials, and potential for scalable production, this approach represents a cost-effective and industrially attractive strategy for constructing three-dimensional porous silicon anodes for next-generation lithium-ion batteries. Despite its advantages, the magnesiothermic reduction route still faces several notable challenges. First, the reaction inevitably generates magnesium oxide as a byproduct, which must be completely removed to avoid residual impurities that can deteriorate electrochemical performance. Second, precise control over reaction kinetics is essential, as uniform porosity and reproducible product quality strongly depend on carefully optimized temperature, time, and magnesium distribution.

To address these challenges and enable the sustainable production of porous silicon from end-of-life (EoL) photovoltaic (PV) panels, Zhang and co-workers [84] developed a combined ball-milling and alloying/dealloying strategy conducted in molten salt media. In this approach, discarded Si wafers are initially treated with alkaline and acidic solutions to remove major contaminants, including Ag, Al, and SiN_x_ layers. Subsequent mechanical milling yields mesoporous silicon powder (m-Si). The m-Si is then subjected to an electrochemical alloying–dealloying process in molten LiCl–KCl, which simultaneously removes the native oxide layer and induces nanostructuring. Specifically, during the alloying step, Li^+^ ions are electrochemically reduced at the cathode and react with m-Si to form a Li–Si alloy. In the subsequent dealloying step, the Li–Si alloy serves as the anode in the same molten salt electrolyte, releasing Li^+^ back into the melt. This reversible alloying–dealloying process exploits the substantial volume change associated with lithium insertion and extraction, resulting in the formation of porous silicon (p-Si). The obtained p-Si exhibits a significantly higher specific surface area than the precursor m-Si (19.4 m^2^·g^−1^ versus 6.1 m^2^·g^−1^), with a pore size distribution ranging from 2 to 30 nm. Electrode expansion analysis further confirms that the alloying–dealloying strategy effectively generates buffering pore structures. After 200 cycles, the m-Si electrode undergoes a thickness expansion of approximately 236%, whereas the p-Si electrode expands from an initial thickness of 12.4 μm to 23.6 μm, corresponding to a substantially lower expansion of 90.3%. As a result, the p-Si anode demonstrates excellent electrochemical performance, delivering a high specific capacity of 2427.7 mAh·g^−1^ at 1 A·g^−1^ after 200 cycles, with a capacity retention of 91.5% and a remaining capacity of 1383.3 mAh·g^−1^ after 500 cycles. These results highlight molten-salt alloying/dealloying as a promising and scalable strategy for converting EoL PV silicon into high-performance porous anode materials for lithium-ion batteries.

#### 2.4.2. Macroporous and Hierarchical 3D Architectures

Three-dimensional macroporous silicon architectures have emerged as highly attractive anode materials for lithium-ion batteries owing to their ability to simultaneously address mechanical instability and transport limitations. The presence of interconnected macropores provides internal buffering space to accommodate severe volume fluctuations during lithiation and delithiation, while the continuous three-dimensional framework ensures efficient electron transport and electrolyte infiltration, collectively reducing polarization and enhancing electrochemical kinetics.

**Figure 11:**
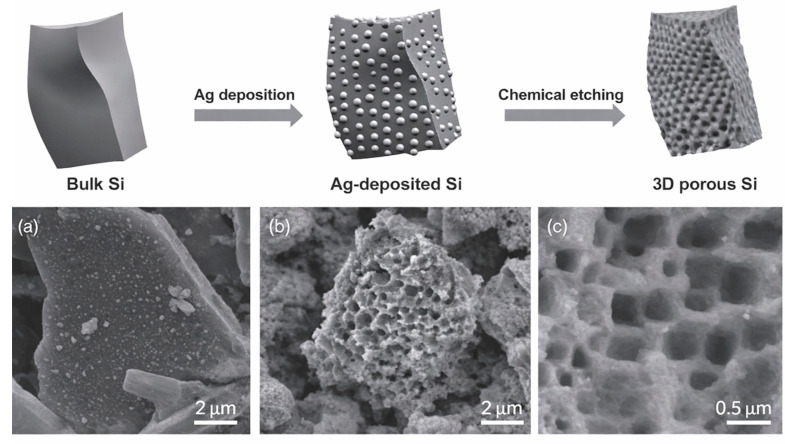
Fabrication and morphological evolution of 3D macroporous silicon via Ag-assisted chemical etching. Upper panel: Schematic of the preparation procedure, showing Ag nanoparticle deposition on bulk Si through a galvanic displacement reaction, followed by Ag-assisted chemical etching to generate interconnected macroporous Si structures. Lower panel: SEM images of (**a**) Ag-deposited Si particles, (**b**) 3D macroporous Si after etching, and (**c**) magnified view highlighting the porous network. Reproduced from Ref. [85], Copyright 2012, with permission from John Wiley and Sons.

Bang et al. [85] synthesized 3D macroporous silicon by combining electroless metal deposition via galvanic displacement with Ag-assisted chemical etching, using commercially available bulk silicon powders as the starting material. As illustrated in Figure 11, Ag nanoparticles deposited on the silicon surface act as localized catalysts that direct anisotropic etching, generating an interconnected macroporous network. This architecture effectively preserves electrical connectivity during cycling and delivers a reversible capacity of approximately 2050 mAh·g^−1^, demonstrating the critical role of macropore connectivity in stabilizing high-capacity silicon anodes.

Similarly, Yang’s research team [86] prepared a highly interconnected 3D macroporous silicon architecture via magnesiothermic reduction, where precise control of reaction parameters enabled the formation of hollow and hierarchical pore structures. Compared with etched macroporous silicon, the magnesiothermic route offers greater flexibility in tailoring pore size distribution and framework continuity. Benefiting from the synergistic effects of macroporosity and carbon modification, the resulting 3D Si@C electrode exhibited excellent cycling stability, retaining a reversible capacity of 1058 mAh·g^−1^ after 800 cycles with a capacity retention of 91%.

Liu and coauthors [87] employed three-dimensional porous silica derived from natural reed leaves as a precursor to fabricate an ultraporous hierarchical SiC architecture via magnesiothermic reduction combined with carbon coating. The resulting 3D SiC framework effectively accommodated large volume variations during cycling while simultaneously facilitating electron and lithium-ion transport, delivering a stable capacity of 420 mAh·g^−1^ after 4000 cycles.

In addition, Yu and coworkers [88] developed silver-coated three-dimensional macroporous silicon via a silver mirror reaction, yielding a highly uniform macroporous structure with pore diameters of approximately 200 nm. This Ag-modified 3D silicon anode demonstrated an exceptionally high discharge capacity of 3585 mAh·g^−1^ along with an initial Coulombic efficiency of 81%, underscoring the effectiveness of conductive surface modification in enhancing electrochemical performance.

Beyond half-cell evaluations, assessing the behavior of three-dimensional silicon-based anodes in full-cell configurations is essential for evaluating their practical relevance. Full-cell studies provide critical insight into electrode compatibility, lithium utilization efficiency, and long-term operational stability under realistic conditions. For example, a representative study [89] reported the synthesis and electrochemical evaluation of Silicon Fuzz@Graphene (SF@G), demonstrating robust cycling performance in both half-cell and full-cell configurations. These results highlight the importance of full-cell validation in bridging laboratory-scale material development with practical lithium-ion battery systems.

While the above examples demonstrate the effectiveness of three-dimensional porous silicon architectures across a range of synthesis strategies and structural designs, their overall performance and applicability are strongly influenced by pore architecture, fabrication routes, and electrode-level trade-offs. A critical comparative analysis of these factors, with particular emphasis on scalability and commercialization considerations, is presented in the following section.

#### 2.4.3. Comparative Analysis and Commercialization Considerations of 3D Porous Silicon Architectures

Three-dimensional porous silicon architectures represent the most structurally balanced class of silicon anodes among the dimensional systems discussed in this review. By integrating interconnected pore networks with continuous solid frameworks, 3D porous silicon effectively reconciles mechanical stress accommodation, electronic conductivity, and lithium-ion transport. Compared with zero- and one-dimensional silicon nanostructures, 3D porous architectures provide enhanced electrode-level robustness, while offering substantially higher areal and volumetric capacities than planar two-dimensional silicon films.

From a comparative perspective, the defining advantage of three-dimensional porous silicon lies in its ability to decouple volumetric expansion from macroscopic electrode deformation. Internal void spaces buffer the large volume changes associated with silicon alloying reactions, thereby suppressing particle fracture, preserving electrical connectivity, and stabilizing the solid electrolyte interphase. Simultaneously, the interconnected 3D framework enables continuous electron and ion transport pathways, mitigating polarization effects that commonly limit the rate capability of dense silicon electrodes. These attributes position porous silicon as a particularly promising candidate for long-life and high-energy lithium-ion battery anodes.

Despite these advantages, the practical implementation of 3D porous silicon architectures remains governed by a fundamental trade-off between porosity and volumetric energy density. Excessive pore volume, while beneficial for buffering silicon expansion, reduces electrode density and increases electrolyte uptake, leading to diminished volumetric capacity and lower initial Coulombic efficiency. Conversely, insufficient porosity compromises mechanical stability and accelerates capacity fading. Achieving an optimal balance between structural buffering and active material loading therefore represents a central challenge in the design of commercially viable 3D silicon anodes.

Manufacturing considerations further complicate large-scale deployment. Many synthesis routes, such as magnesiothermic reduction, metal-assisted chemical etching, and templating strategies, require precise control over reaction kinetics, pore size distribution, and hierarchical organization to ensure reproducible electrochemical performance. Variations in temperature, reductant distribution, or etching uniformity can result in heterogeneous pore architectures and inconsistent cycling behavior. In addition, post-processing steps aimed at removing byproducts or residual templates introduce additional cost and complexity, particularly at industrial scale.

To address these challenges, composite and hybrid design strategies have emerged as essential rather than optional enhancements. The incorporation of carbon coatings, graphene frameworks, or SiO_x_ buffering matrices improves electrical conductivity, suppresses parasitic interfacial reactions, and enhances mechanical resilience during prolonged cycling. Such hybridization strategies enable more precise tuning of pore architecture and electrode density, thereby narrowing the gap between laboratory-scale demonstrations and practical battery requirements.

Beyond half-cell testing, full-cell validation plays a critical role in assessing the true applicability of 3D porous silicon anodes. Full-cell configurations reveal lithium utilization efficiency, electrode balancing constraints, and long-term interfacial stability that cannot be captured in half-cell measurements alone. Recent full-cell studies demonstrate that well-engineered 3D porous silicon electrodes can achieve stable operation with competitive capacity retention, underscoring their potential for real-world lithium-ion battery systems.

Overall, three-dimensional porous silicon architectures offer a compelling compromise between mechanical stability, electrochemical performance, and practical energy density. As summarized in Table 3, a comparative overview of modification strategies for silicon-based anodes across different dimensionalities (0D–3D) highlights the key structure–performance relationships and electrochemical trends associated with dimensional evolution. Continued progress in scalable fabrication, pore architecture optimization, and composite electrode design will be essential for translating 3D porous silicon anodes from advanced laboratory concepts into commercially viable lithium-ion battery technologies.

## 3. Silicon–Carbon Nanocomposites as Stabilization Strategies for Nanostructured Si Anodes

Despite the significant progress achieved through nanoscale and three-dimensional structural engineering of silicon anodes, challenges related to electronic conductivity, SEI instability, and irreversible capacity loss persist. These unresolved issues highlight the necessity of complementary stabilization strategies beyond geometric design alone. Among them, silicon–carbon nanocomposites have emerged as one of the most effective and widely adopted approaches.

In silicon–carbon (Si/C) composite architectures, carbon is introduced as a multifunctional component that simultaneously addresses the electrical, mechanical, and interfacial limitations of silicon anodes [95,96,97]. Owing to its intrinsic flexibility, mechanical robustness, and high electrical conductivity, the carbon phase functions as a compliant buffer matrix that partially accommodates silicon volume expansion while forming continuous electron-transport networks. This dual role alleviates mechanical stress, suppresses electrode pulverization [97], and compensates for the low intrinsic conductivity of silicon [98], thereby reducing internal resistance and stabilizing interfacial reactions with the electrolyte. Consequently, Si/C composites generally exhibit improved cycling stability, enhanced lithiation reversibility, and superior rate performance compared with bare silicon.

From an industrial standpoint, silicon–carbon composites based on Si/graphite blending represent the most mature and commercially viable anode strategy [99]. In current commercial cells, the silicon content is typically limited to 3–10 wt.% to balance capacity enhancement with electrode swelling, mechanical integrity, and cycle life stability. Graphite serves not only as an active material but also as a mechanical buffer and electronic conductor, mitigating the severe volume expansion and low initial Coulombic efficiency associated with silicon. However, increasing the silicon fraction beyond this range often leads to rapid degradation, excessive electrode expansion, and loss of calendaring stability [100], highlighting a fundamental trade-off between energy density and manufacturability.

From a commercialization perspective, different classes of Si/C composites occupy distinct positions along the performance–manufacturability spectrum. Simple Si/graphite blends and carbon-coated silicon powders offer the highest compatibility with existing roll-to-roll electrode manufacturing, slurry processing, and calendaring, but provide only moderate capacity gains due to limited silicon content. In contrast, advanced architectures such as yolk–shell, core–shell, or graphene-supported silicon structures demonstrate superior cycling stability and high specific capacity at the material level, yet face significant barriers related to low tap density, complex multistep synthesis, high carbon content, and poor scalability. CVD-derived Si/C systems further improve interfacial stability and full-cell durability, but their adoption is constrained by precursor cost, reactor throughput, and capital-intensive processing. These trade-offs explain why industrial development currently favors engineered Si powders and moderate-Si blends over more structurally sophisticated nanocomposites. To clarify these performance–manufacturability trade-offs, Table 4 provides a comparative overview of representative Si/C composite architectures, highlighting their typical silicon content, key electrochemical advantages, principal limitations, and relative commercialization readiness.

Various structural designs have been proposed to exploit the synergistic effects between silicon and carbon components. Du and colleagues [101] prepared a core–shell Si@C nanocomposite by covalently grafting aniline monomers onto the silicon surface followed by carbonization. The uniform and elastic carbon coating effectively enhanced electronic conductivity and accommodated volume variations of silicon particles, enabling the electrode to retain a reversible capacity exceeding 750 mAh·g^−1^ after 100 cycles, which was significantly superior to that of pristine silicon.

**Figure 12:**
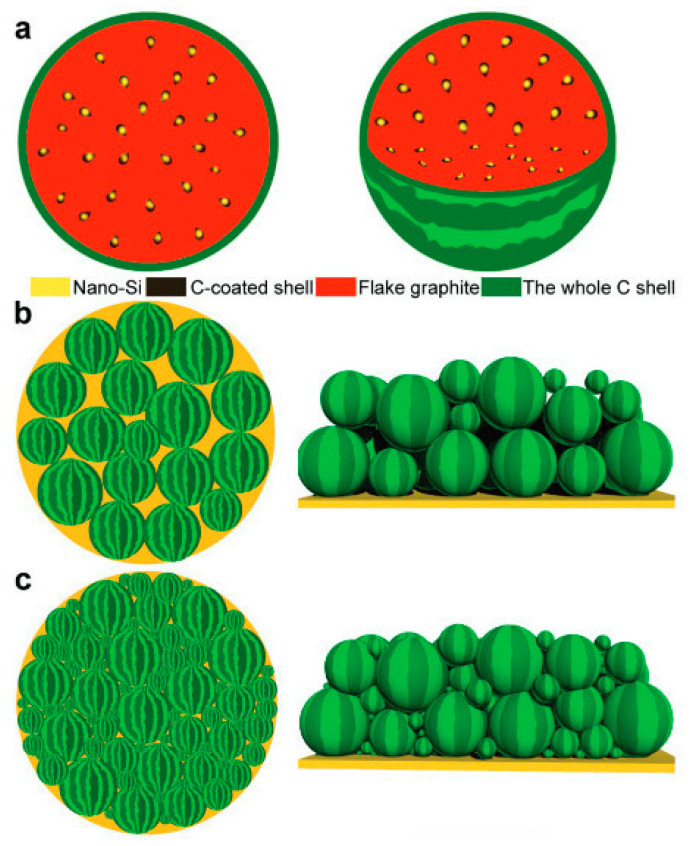
Watermelon-inspired hierarchical Si/C microspheres and their packing models. (**a**) Schematic illustration of the internal buffer structure of the Si/C microspheres. (**b**) Packing model with single-size distribution. (**c**) Optimized packing model with varied microsphere sizes for efficient space occupation and improved mechanical support. Reproduced from Ref. [102], Copyright 2017, with permission from John Wiley and Sons.

Xu and co-workers [102] reported a hierarchical “watermelon-like” Si/C microsphere featuring an internal buffer structure and optimized particle size distribution. At a current rate of 0.1 C, the Si/C anode delivered discharge and charge capacities of 695 and 620 mAh·g^−1^, respectively, with an initial Coulombic efficiency of 89.2%. Owing to its high tap density and structural robustness, the electrode maintained a reversible areal capacity above 1.91 mAh cm^−2^ after 500 cycles. Moreover, the Si/C microspheres exhibited stable cycling and rate performance over a wide temperature range (−20, 25, and 55 °C), retaining approximately 80% of the initial charge capacity even at a high rate of 5 C. The hierarchical structure and optimized size distribution of these “watermelon-like” Si/C microspheres are schematically illustrated in Figure 12. The figure highlights the internal buffer regions, carbon coatings, and packing arrangements, which collectively enable high tap density, efficient stress accommodation, and stable cycling performance. The excellent electrochemical behavior was attributed to the presence of internal pores that effectively buffered silicon volume expansion, reduced stress accumulation, and preserved electrode integrity during prolonged cycling.

More sophisticated architectures have also been developed to further enhance interfacial stability. Xie and collaborators [103] designed a core–shell–yolk–shell Si@C@void@C nanostructure, in which carbon-coated Si nanoparticles served as the yolk encapsulated within a hollow carbon shell. Compared with conventional Si@void@C structures, the additional carbon shell reduced interfacial resistance between the silicon core and the outer carbon shell while simultaneously protecting the silicon from electrolyte corrosion. Consequently, the Si@C@void@C electrode exhibited a high initial reversible capacity of 1910 mAh·g^−1^ at 100 mA·g^−1^ with a capacity retention of 71%, and maintained a capacity of 1366 mAh·g^−1^ after 50 cycles at 500 mA·g^−1^.

Beyond conventional carbon coating strategies, advanced silicon–carbon composites have been developed using chemical vapor deposition (CVD). In these approaches, resin-derived or bio-based porous carbons serve as carbon matrices, while silanes act as silicon precursors. Silicon deposition proceeds via sequential adsorption and thermal decomposition within the porous carbon framework, followed by secondary carbon coating using gaseous carbon sources such as acetylene or ethylene. Sung and co-workers [104] employed ethylene to suppress silicon crystal growth and synthesized subnanometer silicon embedded in a dual matrix of silicon carbide and amorphous carbon via CVD. The resulting C(5)Si–G electrode demonstrated excellent electrochemical performance, achieving a Coulombic efficiency of 99.96% after 50 cycles at 0.1 C. Notably, a 110 A·h full cell assembled with this material exhibited outstanding durability, retaining 91% of its capacity after 2875 cycles and 97.6% after 365 days of calendar aging. The superior performance was attributed to the robust carbon framework that stabilized subnanometer silicon, effectively buffered volume changes, and suppressed unfavorable phase evolution through the formation of stable Si–C bonds.

Despite their outstanding electrochemical properties, CVD-derived Si–C materials still face significant challenges in large-scale industrial implementation, including the high cost of silane precursors, stringent requirements for porous carbon substrates, and complex deposition processes. Further optimization of equipment design and process scalability is therefore required to enable continuous and cost-effective manufacturing of advanced Si–C composite anodes.

Beyond chemical composition, the electrochemical behavior of Si/C anodes is strongly influenced by the dimensionality and spatial organization of the carbon architecture, which links carbon design directly to the silicon nanostructures discussed in the previous sections. Overall, representative silicon–carbon composite anodes reported in the literature demonstrate substantially improved cycling stability and electrochemical performance, particularly for architectures employing three-dimensional carbon frameworks, which effectively buffer silicon volume changes and maintain structural integrity during long-term cycling.

## 4. Key Challenges and Limitations of Nanostructured Silicon Anodes

Despite remarkable progress in the design of nanostructured silicon anodes, several critical challenges continue to impede their large-scale adoption in commercial lithium-ion batteries. One of the most persistent issues is the irreversible loss of lithium during cycling. The high surface area of silicon nanostructures accelerates the continuous formation and regeneration of the solid electrolyte interphase, consuming lithium ions and contributing to initial capacity loss. While strategies such as carbon coatings, porous 3D architectures, and Si–C composite designs can mitigate SEI growth, achieving complete suppression of irreversible lithium consumption remains a significant hurdle.

Scalability is another key limitation. Advanced silicon morphologies, including two-dimensional thin films, three-dimensional porous frameworks, and CVD-derived silicon–carbon composites, often rely on low-throughput or costly synthesis techniques. Translating laboratory-scale fabrication into reproducible, high-yield, and industrially viable processes is challenging, particularly when maintaining precise nanostructural features is essential for performance.

The intrinsic trade-off between structural accommodation and volumetric energy density also presents a challenge. Highly porous or hollow silicon architectures are effective at buffering the substantial volume changes during lithiation and delithiation, but the resulting low electrode density limits the achievable volumetric energy, a critical parameter for electric vehicles and high-energy-density applications. Moreover, the fabrication of mechanically robust silicon electrodes requires carefully optimized slurries with suitable binders and conductive additives. Suboptimal formulations can lead to poor adhesion, loss of electrical contact, and accelerated capacity degradation under repeated cycling.

Thermal stability and safety are further concerns. Nanoscale silicon exhibits high chemical reactivity, which can lead to localized heating and potential thermal runaway under high-rate cycling or abusive conditions. Protective strategies, including carbon coatings, hybrid Si–graphite designs, and electrolyte additives, enhance stability, but intrinsic risks associated with silicon’s reactivity remain non-negligible.

Industrial approaches to mitigate these challenges are increasingly focused on hybrid Si–C anode systems, which currently represent the most realistic pathway toward commercialization. In practical electrode designs, Si/graphite blended anodes, in which the silicon content is typically limited to 3–10 wt.%, are widely adopted to balance capacity enhancement with acceptable electrode swelling, calendaring stability, and cycle life. While this approach improves manufacturability and safety, the achievable capacity gain remains modest, and further increases in silicon content often lead to rapid degradation and excessive electrode expansion.

More advanced Si–C architectures, such as core–shell or yolk–shell Si@C particles and graphene- or CNT-supported silicon frameworks, demonstrate excellent cycling stability and high specific capacity in half-cell configurations. However, their translation to commercial cells is hindered by low tap density, complex multistep synthesis routes, high material cost, and limited compatibility with conventional roll-to-roll electrode manufacturing. In addition, many of these systems still suffer from low initial Coulombic efficiency, necessitating prelithiation strategies that add further complexity at the cell level.

Scalable carbon-coated silicon powders and porous Si–C composites offer a practical compromise between electrochemical performance and manufacturability. Nevertheless, achieving consistent quality, impurity control, and reproducible pore architecture at industrial scale remains challenging. As a result, the commercialization of nanostructured silicon anodes is governed not only by intrinsic electrochemical performance, but also by process scalability, cost per kilowatt-hour, electrode density, and long-term reliability under realistic operating conditions.

## 5. Future Research Directions and Emerging Strategies

Addressing the challenges of nanostructured silicon anodes requires innovative materials design, scalable fabrication techniques, and advanced characterization methods. Promising research directions include:

- Si–graphite hybrid anodes—Integrating silicon with graphite combines the high specific capacity of silicon with the mechanical stability and high volumetric density of graphite, offering a practical route for scalable, high-energy anodes.
- Pre-lithiation techniques—Methods such as stabilized lithium metal powders (SLMP) or electrochemical prelithiation compensate for initial lithium loss associated with SEI formation, improving first-cycle Coulombic efficiency.
- Solid-state silicon-based anodes—Incorporating silicon into solid electrolytes addresses SEI instability and enhances safety, enabling stable cycling at high voltages and temperatures.
- Advanced Si–C composites—Hierarchical and core–shell carbon coatings, conductive networks, and porous 3D frameworks further buffer volume changes, maintain structural integrity, and enhance electron transport. Scalable methods, including chemical vapor deposition (CVD) and magnesiothermic reduction with carbon integration, are key for industrial adoption.
- Sustainable silicon sources—Recycling end-of-life photovoltaic wafers or deriving silicon from biomass offers environmentally friendly and cost-effective alternatives to conventional silicon precursors, aligning with circular economy principles.
- Machine-learning-assisted materials discovery—Computational approaches can accelerate the design of silicon composites, optimize carbon coatings, and predict electrochemical performance, reducing experimental trial-and-error.
- Operando characterization techniques—In situ spectroscopy and microscopy enable real-time monitoring of SEI evolution, volume changes, and degradation mechanisms, providing critical insights for rational electrode design.

By combining these strategies, future research aims to overcome the intrinsic limitations of nanostructured silicon anodes, achieving a balance between high capacity, long-term cycling stability, volumetric energy density, and manufacturability. These advances will be crucial for translating laboratory-scale innovations into commercially viable lithium-ion battery technologies, consistent with the focus of “Nanostructured Silicon Anodes for Lithium-Ion Batteries: Advances, Challenges, and Future Prospects.”

## 6. Conclusions

Nanostructuring has fundamentally transformed the development of silicon anodes for lithium-ion batteries. Engineering sub-10 nm nanoparticles, porous frameworks, hollow structures, and silicon–carbon composites has significantly alleviated the major mechanical and chemical degradation mechanisms associated with silicon, including severe volume expansion and unstable SEI formation. These advances have enabled higher capacity retention, improved rate capability, and prolonged cycling stability compared with conventional bulk silicon and graphite anodes.

Despite this progress, several challenges remain. High production costs, low electrode density, and difficulties in large-scale manufacturing continue to hinder the widespread commercialization of nanostructured silicon anodes. Additionally, achieving consistent performance in full-cell configurations, mitigating irreversible lithium loss, and ensuring thermal and electrochemical safety remain critical concerns.

Ongoing innovations in material design, interface engineering, and electrolyte formulation are addressing these limitations. Strategies such as hierarchical 3D porous architectures, advanced Si–C composite coatings, Si–graphite hybrids, and sustainable silicon sources from biomass or recycled waste are showing promising results. Coupled with machine-learning-assisted optimization and operando characterization techniques, these approaches accelerate the rational design of high-performance silicon anodes.

Future progress will increasingly depend on integrating nanostructured silicon into practical electrode architectures that balance high specific and volumetric capacities with mechanical robustness and manufacturability. Scalable synthesis routes, improved electrode formulations, and compatibility with both liquid and solid electrolytes are essential to translate laboratory-scale advances into commercial lithium-ion battery systems. Collectively, these developments highlight the potential of nanostructured silicon anodes to drive the next generation of high-energy, long-life batteries.

From a practical application perspective, the near-term impact of nanostructured silicon anodes is most likely to be realized through incremental integration rather than full silicon replacement. In electric vehicle batteries, hybrid Si–graphite and Si–C anodes with moderate silicon content are expected to enable measurable gains in gravimetric energy density while maintaining acceptable cycle life, safety, and compatibility with existing manufacturing infrastructure. Such systems are particularly attractive for extending driving range without fundamentally altering cell design or production lines.

For large-scale energy storage applications, where cost, safety, and long-term durability outweigh volumetric energy density, nanostructured silicon is more likely to be employed in mechanically stabilized composite architectures or low-silicon-content hybrids that prioritize cycling stability and thermal robustness. In this context, sustainable silicon sources, scalable synthesis routes, and simplified electrode formulations will play a decisive role.

Overall, the successful translation of nanostructured silicon anodes into real-world applications will depend not only on material-level performance but also on electrode engineering, manufacturing compatibility, and system-level optimization. Continued convergence of materials science, process engineering, and cell design is expected to position nanostructured silicon as a key enabler of next-generation lithium-ion batteries for both electric mobility and stationary energy storage.

## Figures and Tables

**Table 1:** Comparative Overview of 0D Silicon Nanoparticle Fabrication Methods.

Method	Scalability	Cost	Practical Challenges	Industrial Relevance	Main Commercialization Barriers
Ball Milling	High (kg–ton scale)	Low	Agglomeration, partial amorphization, requires carbon integration	Widely used; suitable for recycled Si and bulk feedstocks	Control of particle uniformity, SEI formation, and initial Coulombic efficiency
Chemical Etching	Medium (batch process)	Medium	Hazardous HF, safety concerns, limited throughput	Mainly lab/pilot-scale	Safety, environmental concerns, limited scale-up
Self-Assembly/EISA	Medium–Low	High	Expensive block copolymers, complex processing	Suitable for specialized composites	High cost, difficult scale-up, batch variability
Hydrothermal	Medium	Medium	Long reaction times, energy-intensive, pressure vessels needed	Limited industrial applicability	Energy consumption, slow throughput, reactor complexity
Magnesiothermic Reduction	Low–Medium	Medium–High	HF post-treatment, risk of overheating, complex post-processing	Challenging for large-scale, promising for waste-derived Si	Process complexity, safety, scalability, cost
CVD/Yolk–Shell Coatings	Low	High	High-temperature, vacuum equipment, costly	Mostly lab-scale	Expensive, equipment-intensive, difficult to scale

**Table 2:** Comparative Overview of 1D Silicon Nanostructure Fabrication Methods.

Method	Scalability	Cost	Technical Challenges	Industrial Relevance	Key Trade-Offs/Barriers
VLS/CVD	Low	High	Requires metal catalysts, high temperatures, uniformity control	Low	Excellent control over diameter and crystallinity, but expensive and difficult to scale
Molten salt electrolysis	Medium	Medium	Uniformity and crystallinity control, high-temperature operation	Medium	Green, low-cost, and scalable potential; still developing for uniform large-scale production
Template-assisted/hard-templating	Low	High	Multistep synthesis, template removal, reproducibility	Low	High structural precision and hollow morphologies, but complex and low-throughput
Electrochemical/chemical etching	Medium	Low	Etchant handling, reproducibility, morphology control	Medium	Moderate cost, scalable, but less precise diameter and crystallinity control

**Table 3:** Comparative overview of modification strategies for silicon-based anodes and their electrochemical performance.

Electrode Modification Strategies	Sample/Approach	The Electrochemical Properties	Refs, Year
0D Si Nanostructuring	Si@HC/CNFs (silicon nanoparticles encapsulated in hollow carbon-coated carbon nanofibers)	Initial discharge/charge capacities of 3731.6/1995.2 mAh·g^−1^ at 0.2 A·g^−1^ (ICE = 53.4%); reversible capacity of 1020.7 mAh·g^−1^ after 100 cycles at 0.2 A·g^−1^, corresponding to a capacity retention of 51.2%; electrode mass loading ~2.6 mg·cm^−2^; areal capacity of 3.25 mAh·cm^−2^ at 0.5 mA·cm^−2^.	[90], 2017
0D Si Nanostructuring	Si/C-CNFs (silicon nanoparticles anchored on carbon nanofiber framework)	Initial discharge/charge capacities of 1940/1222.7 mAh·g^−1^ (ICE = 63%); a discharge capacity of 1215.2 mAh·g^−1^ at 0.6 A·g^−1^ after 50 cycles, corresponding to a capacity retention of 99.4%. Mass loading and areal capacity not reported.	[91], 2015
0D Si Nanostructuring	PV panel–derived Si nanoparticles	High specific capacity of 1086.6 mAh·g^−1^ (62.3% of the initial capacity) after 500 cycles at 1.0 C, with a coulombic efficiency above 99%. Electrode mass loading and areal capacity not explicitly reported.	[37], 2023
0D Si Nanostructuring	Industrial fly ash–sourced Si NPs via magnesiothermic method	Initial discharge/charge capacities of 3651.1/3173.1 mAh·g^−1^ at 0.1 C (ICE = 86.9%); reversible capacity of 1030.4 mAh·g^−1^ after 500 cycles at 0.36 A·g^−1^. Electrode mass loading: 0.8–1.5 mg·cm^−2^; areal capacity not reported.	[35], 2022
0D Si Nanostructuring	PV panel–derived Si/graphite hybrid anode	Charge capacity of 426 mAh g^−1^ after 600 cycles (70% retention); rate capability of 215 mAh g^−1^ at 5 C; average coulombic efficiency of 99.4%. Electrode mass loading and areal capacity not reported.	[36], 2021
0D Si Nanostructuring	PV cell–recovered Si nanoparticles produced by mechanical milling	1285 mAh·g^−1^ after 50 cycles with high-capacity retention due to stable SEI formation. Electrode mass loading and areal capacity not reported.	[34], 2020
0D Si Nanostructuring	Si@C nanoparticles obtained by Mg reduction	1756 mAh·g^−1^ after 500 cycles at 2.1 A·g^−1^ (highly stable, comparable to state-of-the-art Si anodes). Electrode mass loading: ~1.0 mg·cm^−2^ (anode); 16 mg·cm^−2^ (cathode, full cell); areal capacity not reported.	[38], 2017
One-dimensional Si Nanostructuring (binder-free nanowire network)	Nanobranched In-seeded Si NW networks grown on copper-silicide current collector	Stable cycling for over 900 cycles with an average coulombic efficiency above 99.6% and a capacity retention of 88.7%; volumetric capacity of 1086.1 mAh·cm^−3^ at 5 C; full-cell capacity of 1177.1 mAh·g^−1^ at 1 C versus LMO. Mass loading of 1.04 mg·cm^−2^; areal capacity of 1.9 mAh·cm^−2^ after extended cycling.	[73], 2023
One-dimensional Si Nanostructuring (Si nanowires) + graphene-based composite	Silicon nanowires embedded in reduced graphene oxide (SiNWs@RGO)	Ultrahigh initial coulombic efficiency of 89.5%; high reversible capacity of 2381.7 mAh·g^−1^ at 1 A·g^−1^ maintained for over 500 cycles with robust cycling stability at a Si content of 76%. Electrode mass loading: ~3.67 mg·cm^−2^; areal capacity not reported.	[52], 2021
One-dimensional Si Nanostructuring	30 nm ultrathin silicon nanowires (UTSiNWs)	High reversible capacity of 933.1 mAh·g^−1^ after 50 cycles at 0.3 A·g^−1^, corresponding to a capacity retention of 87.5% due to stable SEI formation. The electrode mass loading and areal capacity were not reported.	[51], 2020
One-dimensional Si Nanostructuring (Si nanowires) + graphite composite	Carbon-coated silicon nanowires/graphite composite (SiNWs/G@C)	Initial discharge/charge capacities of 808.9/674.4 mAh·g^−1^ at 0.5 C (ICE = 83.37%); reversible capacity of 608.5 mAh·g^−1^ after 100 cycles at 0.5 C, corresponding to a capacity retention of 90.04%. Neither electrode mass loading nor areal capacity is provided.	[50], 2020
One-dimensional Si Nanostructuring (nanotubes)	Sealed Si nanotubes	Initial discharge/charge capacities of 2924/2645 mAh·g^−1^ at 0.2 C (ICE ≈ 90%); reversible capacity of 2169 mAh·g^−1^ after 50 cycles, corresponding to a capacity retention of ~81%. Mass loading: 0.266 mg·cm^−2^; areal capacity: ~0.40 mAh·cm^−2^ (after 50 cycles).	[92], 2013
One-dimensional Si Nanostructuring	Silicon nanotubes	Initial discharge/charge capacities of 3648/3247 mAh·g^−1^ at 0.2 C (ICE = 89%); capacity retention of 89% after 200 cycles at 1 C. Mass loading and areal capacity not reported.	[56], 2009
Two-dimensional Si Nanostructuring (silicon nanosheets) + graphene composite	Ultrathin silicon nanosheets hybridized with reduced graphene oxide (Si-NSs@rGO)	Ultrahigh rate capability of 2395.8 mAh·g^−1^ at 0.05 A·g^−1^ and 1727.3 mAh·g^−1^ at 10 A·g^−1^; ultrastable cycling with a reversible capacity of 1006.1 mAh·g^−1^ at the 1000th cycle (2 A·g^−1^), with Coulombic efficiency reaching 99.5% after two cycles and averaging 99.85% over 1000 cycles; capacity retention enhanced to 84.2% due to graphene hybridization. Mass loading and areal capacity not reported.	[67], 2022
Two-dimensional Si Nanostructuring (porous sandwich-like nanosheets)	Carbon-coated Si/rGO/Si sandwich nanosheets (C/Si–rGO–Si/C)	High reversible capacity of 1187 mAh·g^−1^ after 200 cycles at 0.2 A·g^−1^; excellent cycle stability with 894 mAh·g^−1^ after 1000 cycles at 1 A·g^−1^, corresponding to 70.3% capacity retention and 0.03% capacity decay per cycle; high-rate capability of 694 mAh·g^−1^ at 5 A·g^−1^ and 447 mAh·g^−1^ at 10 A·g^−1^. Mass loading: ~1.0 mg·cm^−2^; areal capacity: ~1.2 mAh·cm^−2^ (after 200 cycles).	[72], 2017
Two-dimensional Si Nanostructuring (multilayer silicene)	Multilayer silicene/graphite composite	Stable reversible capacity of ∼290 mAh·g^−1^ at 1 C after 500 cycles with Coulombic efficiency > 97% and capacity retention > 93%. Mass loading and areal capacity not reported.	[66], 2023
Two-dimensional Si Nanostructuring (sheet-stacked Si/C)	Sheet-stacked silicon/carbon composite	Reversible capacity of 693 mAh·g^−1^ after 300 cycles at 1.0 A·g^−1^, demonstrating stable cycling performance of the sheet-stacked Si/C composite anode. Mass loading: ~0.65–0.85 mg·cm^−2^; areal capacity not reported.	[68], 2022
Two-dimensional Si Nanostructuring (carbon-coated nanosheets)	Carbon-coated silicon nanosheets (C-SiNSs)	High reversible capacity of ~2100 mAh·g^−1^ at the 300th cycle (0.15 C) with approximately 100% Coulombic efficiency, demonstrating excellent cycling stability. Mass loading: ~6.2 mg·cm^−2^; areal capacity: 3.02 mAh·cm^−2^ (after 100 cycles).	[70], 2021
Two-dimensional Si Nanostructuring (porous nanosheets)	Porous silicon nanosheets synthesized on graphene oxide template (Si-NSs)	Reversible capacity of 800 mAh·g^−1^ after 900 cycles at 8.4 A·g^−1^, demonstrating high-rate stability and structural retention due to the mesoporous nanosheet architecture. Mass loading: 0.8 mg·cm^−2^; areal capacity: not reported (estimated ~0.64 mAh·cm^−2^).	[71], 2019
Three-dimensional Si Nanostructuring (porous SiO_x_/nanoSi with carbon coating)	0.75SiO_x_/0.25nanoSi@C composite	Reversible capacity of 1067.92 mAh·g^−1^ after 400 cycles at 1 A·g^−1^, demonstrating high cycling stability due to the 3D porous architecture and carbon coating. Mass loading: ~0.5 mg·cm^−2^; areal capacity: not reported (estimated ~0.53 mAh·cm^−2^).	[83], 2023
Three-dimensional Si Nanostructuring (porous Si from recycled PV panels)	Porous Si produced via combined ball-milling and alloying/dealloying of end-of-life PV panels	Discharge capacity of 2427.7 mAh·g^−1^ at 1 A·g^−1^ after 200 cycles, with 91.5% capacity retention (1383.3 mAh·g^−1^ after 500 cycles), demonstrating high cycling stability of the 3D porous structure. Mass loading: 0.5~0.9 mg·cm^−2^; areal capacity not reported.	[84], 2021
Three-dimensional Si Nanostructuring (porous Si coated with nitrogen-doped carbon)	Porous silicon–carbon nanocomposite (pSi@NC)	Initial discharge/charge capacities of 3057/2182 mAh·g^−1^ (ICE = 71.4%); retained discharge capacity of 1300 mAh·g^−1^ after 200 cycles at 1 A·g^−1^; 750 mAh·g^−1^ at 2 A·g^−1^ after 250 cycles; Coulombic efficiency ≈100% throughout cycling. Mass loading: 0.5 mg·cm^−2^; areal capacity: not reported (estimated ~0.65 mAh·cm^−2^).	[93], 2019
Three-dimensional Si Nanostructuring (porous nanoparticle network, binder-free)	EPD-5s: binder-free Si nanoparticle electrode with 3D porous structure prepared by 5 s electrophoretic deposition	Discharge capacity of 1913 mAh g^−1^ after 100 cycles at 0.1 C, with a capacity retention of 73% and Coulombic efficiency reaching 97–98% during cycling. Mass loading: 0.25–1.10 mg·cm^−2^; areal capacity: up to ~1 mAh·cm^−2^.	[94], 2015

**Table 4:** Comparison of representative Si/C composite anodes in terms of electrochemical performance and commercialization readiness.

Si/C Architecture	Typical Si Content	Electrochemical Advantages	Key Limitations	Commercial Readiness
Si/graphite blends	3–10 wt.%	Good cycle life, low swelling, low cost	Limited capacity gain	High
Carbon-coated Si powders	5–15 wt.%	Improved SEI stability, scalable processing	Moderate ICE, coating uniformity	Medium–High
Core–shell/yolk–shell Si@C	20–50 wt.%	Excellent cycling stability, stress buffering	Low tap density, complex synthesis	Medium
Graphene/CNT-supported Si	10–40 wt.%	High conductivity, rate capability	High cost, poor slurry compatibility	Low–Medium
CVD-derived Si–C composites	10–30 wt.%	Outstanding stability, full-cell durability	High precursor cost, low throughput	Low

## Data Availability

No new data were created or analyzed in this study. Data sharing is not applicable to this article.

## Abbreviations

The following abbreviations are used in this manuscript:

LIBs	Lithium-ion batteries
SEI	Solid electrolyte interphase
Si NPs	Silicon nanoparticles
Si NWs	Silicon nanowires
